# Development and Validation of Machine Learning Algorithms to Predict 1-Year Ischemic Stroke and Bleeding Events in Patients with Atrial Fibrillation and Cancer

**DOI:** 10.1007/s12012-024-09843-8

**Published:** 2024-03-18

**Authors:** Bang Truong, Jingyi Zheng, Lori Hornsby, Brent Fox, Chiahung Chou, Jingjing Qian

**Affiliations:** 1 Department of Health Outcomes Research and Policy, Auburn University Harrison College of Pharmacy, 4306d Walker Building, Auburn, AL 36849 USA; 2 Department of Mathematics and Statistics, Auburn University College of Sciences and Mathematics, Auburn, AL USA; 3 Department of Pharmacy Practice, Auburn University Harrison College of Pharmacy, Auburn, AL USA

**Keywords:** AFib, Cancer, Stroke, Bleeding, Prediction, Machine learning

## Abstract

In this study, we leveraged machine learning (ML) approach to develop and validate new assessment tools for predicting stroke and bleeding among patients with atrial fibrillation (AFib) and cancer. We conducted a retrospective cohort study including patients who were newly diagnosed with AFib with a record of cancer from the 2012–2018 Surveillance, Epidemiology, and End Results (SEER)-Medicare database. The ML algorithms were developed and validated separately for each outcome by fitting elastic net, random forest (RF), extreme gradient boosting (XGBoost), support vector machine (SVM), and neural network models with tenfold cross-validation (train:test = 7:3). We obtained area under the curve (AUC), sensitivity, specificity, and F2 score as performance metrics. Model calibration was assessed using Brier score. In sensitivity analysis, we resampled data using Synthetic Minority Oversampling Technique (SMOTE). Among 18,388 patients with AFib and cancer, 523 (2.84%) had ischemic stroke and 221 (1.20%) had major bleeding within one year after AFib diagnosis. In prediction of ischemic stroke, RF significantly outperformed other ML models [AUC (0.916, 95% CI 0.887–0.945), sensitivity 0.868, specificity 0.801, F2 score 0.375, Brier score = 0.035]. However, the performance of ML algorithms in prediction of major bleeding was low with highest AUC achieved by RF (0.623, 95% CI 0.554–0.692). RF models performed better than CHA_2_DS_2_-VASc and HAS-BLED scores. SMOTE did not improve the performance of the ML algorithms. Our study demonstrated a promising application of ML in stroke prediction among patients with AFib and cancer. This tool may be leveraged in assisting clinicians to identify patients at high risk of stroke and optimize treatment decisions.

**Supplementary Information:**

The online version contains supplementary material available at 10.1007/s12012-024-09843-8.

## Introduction

In the United States (US), atrial fibrillation (AFib) is projected to affect 12 million people by 2030 [1]. AFib has been recorded as the primary diagnosis for more than 454,000 hospitalizations and contributed to more than 158,000 deaths annually [2–4]. The coexistence of cancer among patients with AFib increases incidence of adverse events such as ischemic stroke, venous thromboembolism (VTE), bleeding, and death compared with AFib patients without cancer [5–8]. Current management of patients with AFib and cancer with oral anticoagulants (OACs) remains suboptimal due to insufficient evidence regarding risk assessment and treatment optimization from clinical practice guidelines [9].

CHA_2_DS_2_-VASc score, a composite score of congestive heart failure (1 point), hypertension (1), age ≥ 75 (2), diabetes mellitus (1), prior stroke, TIA, or thromboembolism (2), vascular disease (e.g. peripheral artery disease, myocardial infarction, aortic plaque) (1), age 65–74 years, and sex category (1), has been used to evaluate of risk of stroke in patients with AFib [10, 11]. The clinical guidelines recommend OACs for patients with CHA_2_DS_2_-VASc scores ≥ 2 [11, 12]. However, CHA_2_DS_2_-VASc score is not highly predictive in patients with AFib and cancer [13, 14]. HAS-BLED score has been widely used for risk of bleeding stratification. The HAS-BLED is calculated by the presence of hypertension (1), abnormal renal/liver function (1 + 1), stroke (1), bleeding tendency or predisposition (1), labile INR for patients taking warfarin (1), age ≥ 65, drugs (concomitant aspirin or NSAIDs) or excess alcohol use (1 + 1) [15]. The 2020 European Society of Cardiology (ESC) guideline suggests a score of ≥ 3 indicates “high risk” [12]. However, it is not recommend against the use of anticoagulants, but caution and regular monitoring after treatment initiation are needed [12]. Nonetheless, the usefulness of HAS-BLED in cancer patients are inconclusive because cancer is an independent risk factor of bleeding among patients with AFib [16]. Pastori et al. compared the performances of multiple bleeding risk scores among cancer patients and found that HAS-BLED was not highly predictive of major and gastrointestinal bleeding [17].

Therefore, it is an urgent need to develop new risk assessment tools for stroke and bleeding in patients with AFib and cancer. Traditional risk assessment tools such as CHA_2_DS_2_-VASc and HAS-BLED are simple and easy for implementation among clinicians because they are linear combinations of patients’ diseases and conditions. However, when the relationships between patients’ characteristics and outcomes become more complicated, these tools may not perform well in patients with AFib and cancer. Recently, machine learning (ML) algorithms have been increasingly used to support clinical decision-making such as or identifying patients with dementia in primary care, anticoagulation monitoring, and measuring pretreatment quality of care before treatment in patients with hepatitis C [18–20]. Compared with conventional regression-based methods, ML models are able to learn from the data when the association between predictors and outcome variables is not linear. ML models have overperformed parametric regressions in handling high-dimensional data and interactions between variables in a complex data structure [20–22].

In this study, we developed and validated ML algorithms to predict risk of stroke and bleeding events among patients with AFib and cancer, using US cancer registry and administrative claims linked datasets.

## Materials and Methods

### Study Design and Data Source

We followed the Transparent Reporting of a multivariable prediction model for individual Prognosis Or Diagnosis (TRIPOD) guideline to develop and validate ML algorithms to separately predict risk of stroke and risk of bleeding in patients with AFib and cancer [23]. We conducted a retrospective cohort study using the 2011–2019 Surveillance, Epidemiology, and End Results (SEER) registry linked to Medicare database. SEER registry contains demographics, cancer characteristics, treatment, and follow-up of cancer patients across the US, [24] while Medicare data capture health care services utilization (medical claims, procedures, and prescriptions) of beneficiaries [25]. The study design and approach are illustrated in Figures S1, S2.

### Participants

We included individuals aged ≥ 66, newly diagnosed non-valvular atrial fibrillation (NVAF) from 1/1/2012 to 12/31/2018. AFib was defined as any International Classification of Disease-9th Revision-Clinical Modification (ICD-9-CM) codes 427.31 or 427.32 or any International Classification of Disease-10th Revision-Clinical Modification (ICD-10-CM) codes I48.xx in any position on one Medicare inpatient claim or on two outpatient claims at least 7 days but < 1 year apart [26]. We removed patients with valvular diseases, repair or replacement, venous thromboembolism, or joint replacement during the 12 months baseline period because OACs are also indicated for these conditions and their clinical management are different from AFib [27, 28]. Eligible records were then linked to SEER files to identify patients with breast, lung, or prostate cancer—the most common cancer types with AFib—from at any time before the initial AFib diagnosis (ICD-O-3 codes C50.0–C50.9 for breast; C34.0, C34.1, C34.2, C34.3, C34.8, C34.9, C33.9 for lung; C61.9 for prostate cancer). Patients were required to continuously enroll in Medicare part A, B, D, and without Medicare Advantage or Health Maintenance Organization (HMO) for at least 12 months before and 12 months after NVAF diagnosis. Since OAC initiation during follow-up may modify the risk of stroke and bleeding, we excluded patients who initiated warfarin or direct anticoagulants (DOACs) within 12 months before or after NVAF diagnosis. All ICD codes to identify these conditions can be found in Table S1, Supplementary materials.

### Outcomes

The outcomes of interest were ischemic stroke and major bleeding events identified within 12 months after AFib diagnosis. We defined major bleeding and ischemic stroke using validated algorithms defined by ICD-9-CM and ICD-10-CM codes in the primary diagnosis from Medicare medical claims files [29–31].

### Predictors

We selected potential predictors from literature review and based on availability in SEER-Medicare data [29, 32, 33]. The following predictors were included: *demographics* (index age, sex, race/ethnicity, calendar year, geographical region, urbanicity), *socioeconomic factors* (household median income, percentage of household with education level below high school, and Medicaid eligibility), *comorbidities* (hypertension, congestive heart failure, diabetes, prior stroke, vascular diseases, prior bleeding, renal diseases, liver diseases, alcohol use disorders, asthma/chronic obstructive pulmonary disease, hematological disorders, dementia, depression, thrombocytopenia, acute kidney disease, peptic ulcer disease), *cancer characteristics* (time from cancer diagnosis to the onset of AFib, cancer type, cancer stage, tumor grade, active cancer status [29, 32]), *cancer treatment* (radiation, and cancer-directed surgery, and potentially interacting antineoplastic agents), and *medication history* (antiplatelet/non-steroidal anti-inflammatory drugs, angiotensin-converting enzyme (ACE) inhibitors/angiotensin II receptor blockers (ARBs), calcium channel blockers, beta blockers, antiarrhythmic medications, diuretics, statin, pump proton inhibitors, and serotonin reuptake inhibitors). Features were obtained during 12 months before the index date. All diagnosis codes and procedure codes for covariate ascertainment are described in Table S1, Supplementary materials.

### Algorithms, Model Training and Validation

Descriptive statistics was used to compare the characteristics of the full cohort and between patients with and without the outcomes. MissForest was used to impute missing values for predictors [34, 35]. The original dataset was then randomly split into two datasets: training (70%) and testing datasets (30%) [36, 37], with similar distribution of the outcomes in both datasets. In the algorithm training process, ML models (elastic net logistic regression, random forest (RF), support vector machine (SVM), extreme gradient boosting (XGBoost), and neural network) were fitted with ten-fold cross-validation (CV) [38]. The fitted models were then tested on the rest of the data. Since stroke and bleeding occurred in less than 10% within one year among AFib patients [29, 39], our classification is severely imbalanced due to the prediction of minority class (stroke and bleeding) [40]. Therefore, we shifted the decision threshold to the true event probability rather than using the default threshold of 0.50 [40, 41]. The description of the models can be found in Technical Appendix.

### Model Performance, Calibration, and Evaluation

To assess algorithm discrimination, we calculated the area under the receiver operating characteristic curve (AUROC or AUC) as the main metrics and compared the AUC across algorithms using DeLong’s test [42]. Other performance metrics were also extracted, including sensitivity, specificity, and F2 score. Since true positive (patients actually having stroke/bleeding) is more important and false negative cases (patients at high risk of the event were not identified) are more costly, we selected F2 score over F1 score [41, 43]. In addition, we generated feature importance plots to identify the contribution of each variable in predicting the outcomes [44]. Since feature importance using Gini index in tree-based algorithms (i.e., RF and XGBoost) are subject to bias [45], we computed out-of-bag impurity reduction feature importance as an alternative [46]. Model calibration was performed to compare the true probability of the outcome versus a model’s prediction with Brier score [47]. We also compared the performances of ML algorithms with CHA_2_DS_2_-VASc score or HAS-BLED score in predicting ischemic stroke and major bleeding, respectively. In predicting ischemic stroke, we fitted logistic regressions with CHA_2_DS_2_-VASc score as the predictor using the training data and validated the model on the testing data. Likewise, we predicted the risk of major bleeding on HAS-BLED score. Sensitivity, specificity, and AUC were obtained for these models. ML algorithms were developed using RStudio (version 3.6.2; Boston, MA, USA) and data analysis was conducted using SAS (version 9.4, SAS Institute, Inc., Cary, NC, USA).

### Sensitivity Analyses

Since our classification problem was severely imbalanced, we used Synthetic Minority Oversampling Technique (SMOTE) to account for imbalance distribution of the outcome variables [48]. Model development and validation were conducted on the resampling dataset.

## Results

### Study Sample and Characteristics

**Fig. 1:**
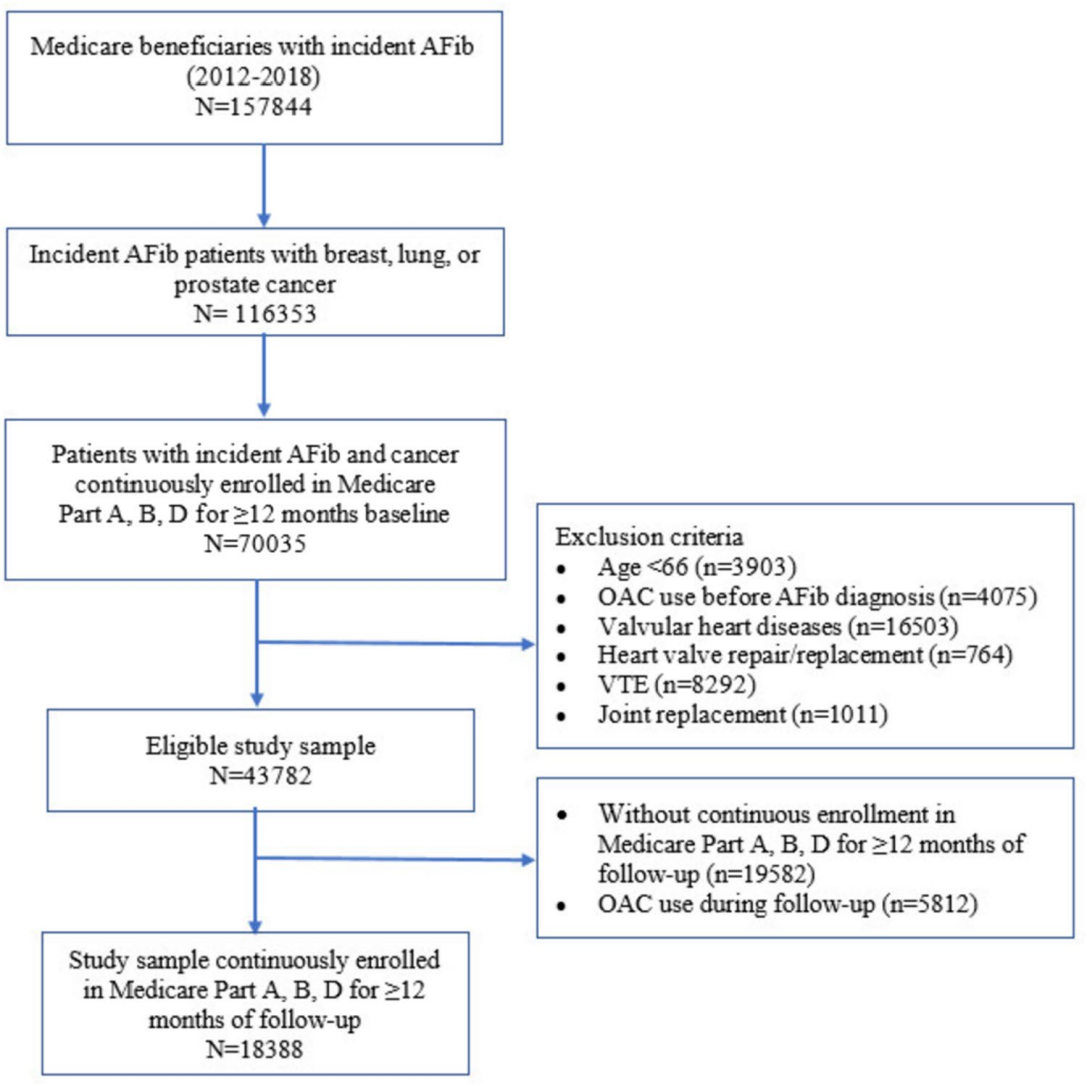
Flowchart diagram for study sample. *VTE* Venous thromboembolism, *AFib* Atrial Fibrillation, *OAC* Oral Anticoagulant

The final cohort consisted of 18,388 patients, of whom 523 (2.84%) had ischemic stroke and 221 (1.20%) had major bleeding within one year after AFib diagnosis (Fig. 1). The characteristics of study sample are described in Table S2. Overall, the mean (standard deviation) age was 76.59 (7.13), 8483 (46.13%) were women, and the majority were White (85.11%) and residing in the Northeast (39.13%), or West (34.40%) region. The median (interquartile range) duration from cancer diagnosis to AFib onset was 17 (2–40) months. Compared with non-stroke patients, patients who had stroke were more likely to have breast cancer [(227 (43.40%) vs. 5416 (30.32%)], use potential interaction agents [139 (26.58%) vs. 825 (21.41%)], diabetes [188 (35.95%) vs. 5433 (30.41)], history of stroke [96 (18.36) vs. 1446 (8.09)], and vascular diseases [136 (26.00) vs. 4249 (23.78)] but less likely to have lung cancer [106 (20.27%) vs 6059 (33.92%)]. Compared with non-bleeding patients, patients who had bleeding were more likely to have breast cancer [84 (38.01%) vs. 5559 (30.60%)], history stroke [53 (23.98%) vs. 1489 (8.20%)], vascular diseases [63 (28.51%) vs. 4322 (23.79%)], and history of bleeding [72 (32.58%) vs. 3771 (20.76%)] (Table S3).

### Algorithm Performance and Comparison

#### Ischemic Stroke Prediction

**Fig. 2:**
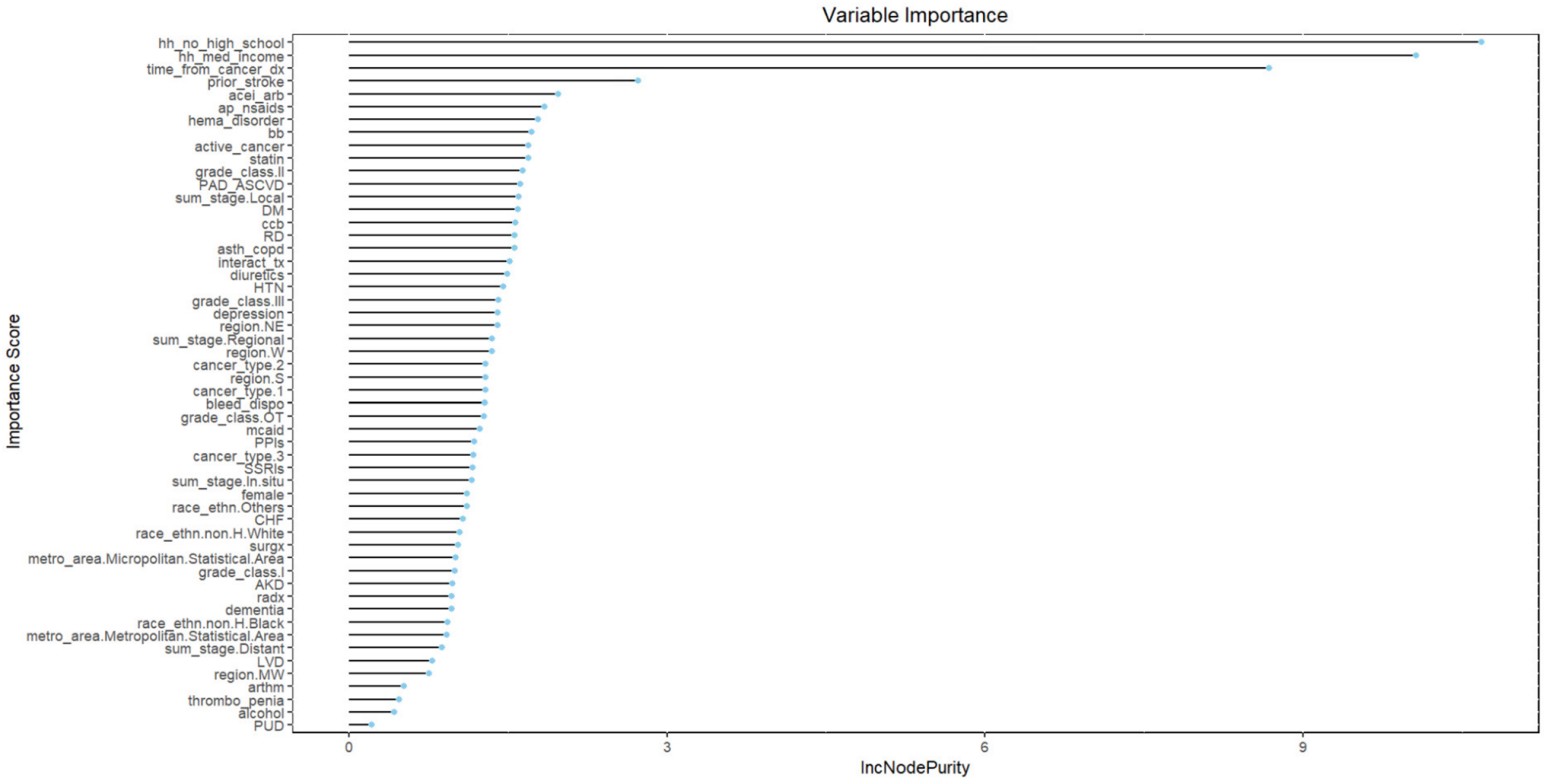
Feature importance plot of random forest algorithm for ischemic stroke prediction (original data)

The performances of ML models in the original sample are described in Table 1. The AUCs of elastic net, RF, XGBoost, SVM, and neural network were 0.684 (95% CI 0.641–0.727), 0.916 (95% CI 0.887–0.945), 0.737 (95% CI 0.698–0.777), 0.545 (95% CI 0.502–0.588), and 0.625 (95% CI 0.579–0.672), respectively. RF outperformed other ML models in AUC (0.916, 95% CI 0.887–0.945), sensitivity (0.868), specificity (0.801), and F2 score (0.375). The best calibration was achieved in RF algorithm (Brier score = 0.035) (Table 1). Although CHA_2_DS_2_-VASc score showed a higher sensitivity (0.829) compared to other ML models (except for RF), its specificity was low (0.268). Top five important features of RF algorithm were socioeconomic factors (proportion of household with no high school education level and median household median income), time from cancer diagnosis to AFib onset, history of stroke, and concomitant use of ACE inhibitors or ARBs. History of stroke and time from cancer diagnosis to AFib onset were the most important features in all ML models (Figs. 2, S3–S6).

**Table 1:** Model performance of machine learning models for ischemic stroke prediction

	Sensitivity	Specificity	AUROC	*p*-value*	F2 score	Brier score
Original data						
Elastic net	0.698	0.574	0.684 (0.641–0.727)	Reference	0.183	0.055
RF	0.868	0.801	0.916 (0.887–0.945)	< 0.001	0.375	0.035
XGBoost	0.723	0.608	0.737 (0.698–0.777)	0.005	0.202	0.054
SVM	0.434	0.589	0.545 (0.502–0.588)	< 0.001	0.121	0.055
NN	0.692	0.511	0.625 (0.579–0.672)	0.023	0.161	0.056
CHA_2_DS_2_-VASc	0.829	0.268	0.580 (0.534–0.623)	–	–	–
SMOTE resampling						
Elastic net	0.577	0.620	0.648 (0.603–0.693)	Reference	0.164	0.446
RF	0.801	0.334	0.633 (0.587–0.675)	0.0352	0.213	0.442
XGBoost	0.667	0.529	0.633 (0.588–0.678)	0.0408	0.160	0.440
SVM	0.560	0.633	0.650 (0.604–0.695)	0.1027	0.172	0.446
NN	0.372	0.752	0.580 (0.534–0.626)	< 0.001	0.152	0.309

AUROC area under receiver operating characteristic curve, RF random forest, XGBoost extreme gradient boosting, SVM support vector machine, NN neural network, SMOTE synthetic minority oversampling technique

*DeLong’s test

–: not calculated

#### Major Bleeding Prediction

**Fig. 3:**
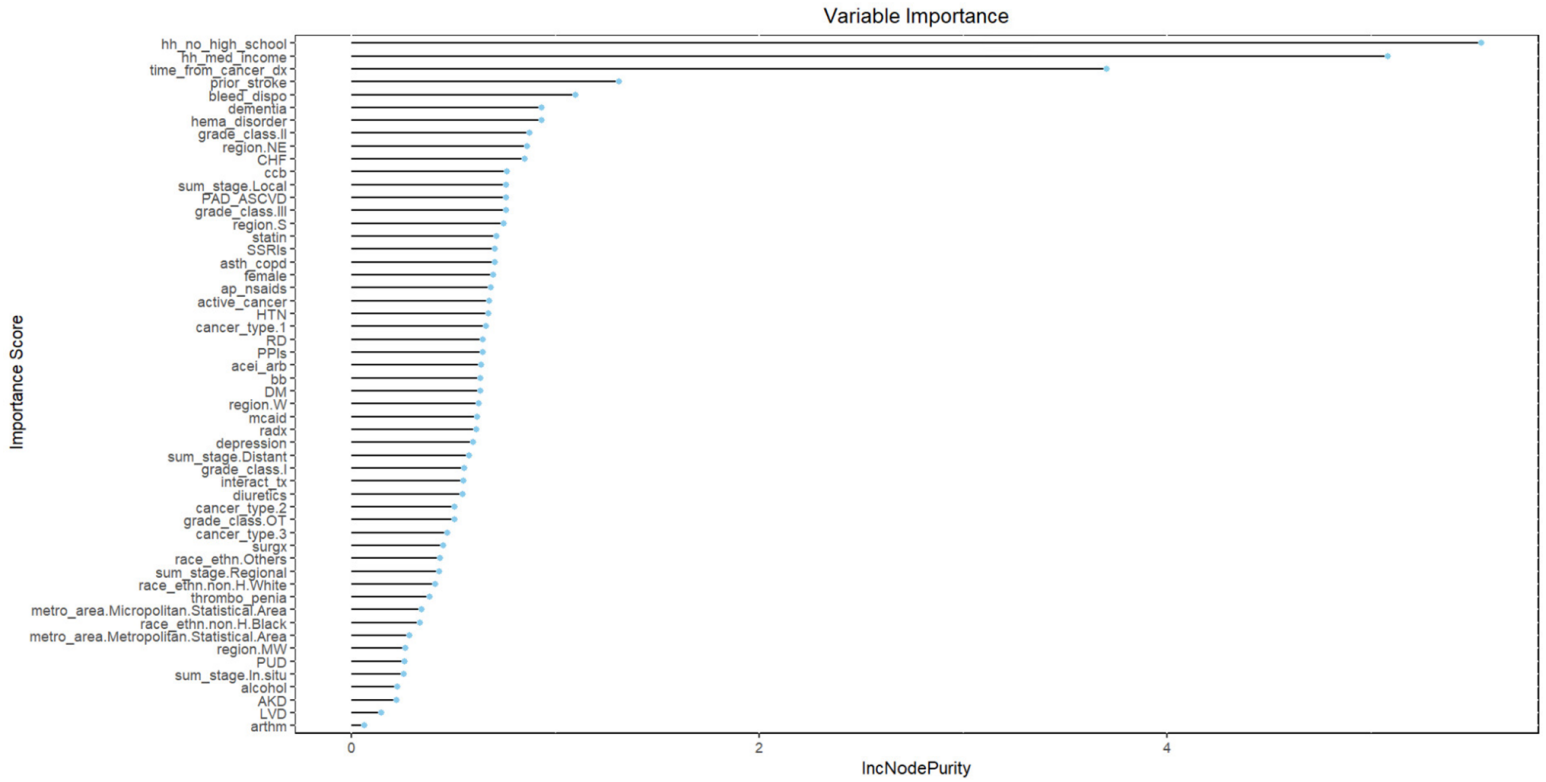
Feature importance plot of random forest algorithm for major bleeding prediction (original data)

For bleeding prediction, performances of ML models were poor (all AUCs < 0.7 in original sample) (Table 2). The AUCs of elastic net, RF, XGBoost, SVM, and neural network were 0.575 (95% CI 0.503–0.649), 0.623 (95% CI 0.554–0.692), 0.578 (95% CI 0.510–0.646), 0.546 (95% CI 0.472–0.619), 0.504 (95% CI 0.432–0.575), respectively. RF outperformed other models in AUC (0.623 (95% CI 0.554–0.692). However, sensitivity was highest for SVM algorithm (0.652), and best specificity was achieved in elastic net algorithm (0.689). There was no difference in calibration of five algorithms. HAS-BLED score failed to identify patients with major bleeding (sensitivity = 0.052). Proportion of household with no high school education level, median household median income, time from cancer diagnosis to AFib onset, history of stroke, history of bleeding were top five important features of RF algorithm. History of bleeding was among top 5 important features in elastic net, RF, XGBoost, and neural network algorithms (Figs. 3**, S7-S10**).

**Table 2:** Model performance of machine learning models for major bleeding prediction

	Sensitivity	Specificity	AUC	p-value	F2	Brier score
Original data						
Elastic net	0.424	0.689	0.575 (0.503–0.649)	Reference	0.070	0.023
RF	0.515	0.671	0.623 (0.554–0.692)	0.0003	0.081	0.024
XGBoost	0.439	0.641	0.578 (0.510–0.646)	0.7210	0.064	0.024
SVM	0.652	0.357	0.546 (0.472–0.619)	0.0726	0.056	0.024
NN	0.470	0.497	0.504 (0.432–0.575)	0.0122	0.051	0.024
HAS-BLED	0.052	0.960	0.574 (0.506–0.637)	–	–	–
SMOTE resampling						
Elastic net	0.348	0.722	0.564 (0.492–0.635)	Reference	0.064	0.378
RF	0.863	0.182	0.551 (0.478–0.625)	0.2752	0.057	0.048
XGBoost	0.515	0.517	0.553 (0.477–0.630)	0.2813	0.057	0.050
SVM	0.348	0.714	0.562 (0.490–0.634)	0.4052	0.062	0.375
NN	0.136	0.849	0.520 (0.449–0.590)	0.2752	0.041	0.200

AUROC area under receiver operating characteristic curve, RF random forest, XGBoost extreme gradient boosting, SVM support vector machine, NN neural network, SMOTE synthetic minority oversampling technique

*DeLong’s test

–: not calculated

### Sensitivity Analysis

There was no major improvement in the performance metrics using SMOTE resampling for ischemic stroke and major bleeding prediction across five ML algorithms. SMOTE resampling worsened the calibration of the algorithms with larger Brier score compared with original sample (Table 1 and 2). Feature importance of ML algorithms in SMOTE samples are described in Figures S11-S15 (ischemic stroke) and Figures S16-20 (major bleeding).

## Discussion

Our study is among the first studies that developed and validated ML algorithms to predict adverse outcomes exclusively for patients with AFib and cancer. In this cohort study, we demonstrated that incorporating ML algorithms into SEER-Medicare data can be a promising tool to predict short-term (1 year) risk of stroke among patients with AFib and cancer. Among older adults with cancer who were newly diagnosed with AFib, clinicians can collect patients’ demographics, socioeconomic status, medical history, and medication history from routine medical records and/or patient survey, then leverage this tool to predict patients’ risk of stroke. Our ML algorithms help clinicians identify high-risk patients and facilitate treatment decision (i.e., medication or non-pharmacological intervention) among older adults with AFib and cancer across the US.

RF outperformed other ML models in all metrics (AUC, sensitivity, specificity, and F2 score) for ischemic stroke. Although widely accepted as a risk assessment tool for stroke among patients with AFib, CHA_2_DS_2_-VASc score failed to achieve high performance in patients with AFib and cancer, especially in new onset AFib [9, 14, 49]. In this study, CHA_2_DS_2_-VASc score performed better than ML models, except for RF in identifying patients with ischemic stroke, however, CHA_2_DS_2_-VASc score could not differentiate those with lower risk (low specificity). In fact, 91.9% of patients in this study have CHA_2_DS_2_-VASc ≥ 2 and would have been recommended for OACs according to current guidelines [11, 12]. The major limitation of CHA_2_DS_2_-VASc is the absence of cancer indicator, which has been suggested as an independent risk factor of stroke [50, 51]. A recently published study suggested the incorporation of cancer to CHA_2_DS_2_-VASc score to improve predictability of the original score [52]. Indeed, CHA_2_DS_2_-VASc score is the linear combination of conditions in prediction of stroke [10]. In the presence of cancer, the relationship between patient characteristics and ischemic stroke may become more complicated (i.e., non-linear), it is not surprising that CHA_2_DS_2_-VASc score failed to achieve high performance. In our study, linear models such as elastic net and SVM had lower performance metrics compared with non-linear models such as RF and XGBoost. Similar to CHA_2_DS_2_-VASc, we found prior stroke was among most important features in all ML algorithms. However, our approach incorporated a comprehensive set of patients’ characteristics. For example, patients’ socioeconomic status (household median income and education level) and cancer characteristics (cancer type, active cancer status) were ranked among top features in RF and XGBoost. The importance of these features highlighted contributions of health disparities and cancer characteristics in stroke prediction. The inclusion of these variables may be useful in identifying high-risk patients [53]. However, it is also noticed that tree-based models may inflate the impact of continuous features in their prediction [45]. Clinicians may consider initiating OACs for those who are at high risk of stroke identified by our RF algorithm.

Traditional tools such as HAS-BLED or HEMORR_2_HAGES showed poor predictability in patients with cancer [16, 17, 54]. Our ML algorithms also failed to obtain high performance metrics in prediction of major bleeding. Such poor performance suggested complex interactions between patients’ characteristics and outcomes in the presence of cancer. First, although we obtained additional cancer characteristics compared with traditional risk scores, the performance was not improved [55]. This may suggest that our models failed to capture important features in prediction of major bleeding. In fact, genetic factors and disease severity were not available in SEER-Medicare data and dynamic features (i.e., cancer progression, new diagnosis of diseases) were not included in the models due to complexities. Similar to previous risk scores, we found that bleeding history was an important factor in prediction of subsequent major bleeding [17, 55]. Second, we excluded patients who have already initiated OACs before AFib diagnosis and those who initiated AFib during follow-up because OACs may increase risk of bleeding. As a result, only 1.2% patients in our cohort experienced bleeding events during follow-up and this created a severe imbalance classification problem for our ML algorithms and may lead to poor predictability [56]. Future studies may expand the outcomes to other types of bleeding (i.e., intracranial bleeding, gastrointestinal bleeding, or other non-critical site bleeding) to improve the performance and the clinical utility of the algorithms.

In our study, SMOTE resampling approach did not improve the performance of the model. In the training set, SMOTE created new synthetic ‘stroke’ individuals from interpolations of the original, real ‘stroke’ cases [48]. Studies have shown that SMOTE-like methods could improve the performance of weak classifiers such as SVM, decision tree [57]. In our study, SMOTE improved AUCs in SVM only. Another limitation of SMOTE is that it resulted in poorly calibrated models where the probability of the minority class (stroke) was strongly inflated demonstrated by Brier score.

Our study is subject to some limitations. We were unable to capture some important variables in the ML models (i.e., BMI, genetic factors, frailty, and health behaviors—not available in SEER-Medicare). Socioeconomic factors such as household income and education level are available on the aggregate area level (Census tract) but not individual level. In addition, our algorithms did not incorporate the impact of some post-baseline predictors (i.e., treatment dosage, adherence, recent CHA_2_DS_2_-VASc and HAS-BLED scores, recent use of NSAIDs, and other time-varying variables such as interactions between oral anticoagulants between OACs and antineoplastic agents) [58]. Our study is applicable to the study period 2011–2019. From 2020, the presence Covid-19 has worsened outcomes of patients with AFib or cancer patients and has negatively impacted health services, delayed and reduced cancer screening and diagnosis in the United States [59–63]. Therefore, the model should be updated and validated incorporating Covid-related factors during and after the pandemic. In addition, our ML algorithms could not further stratify the risk of stroke and major bleeding (i.e., low, moderate, high, or very high). Future study may leverage advanced ML algorithms such as survival ML in predicting the probability of adverse events after 1 year or extended follow-up time. Last, the generalizability of our ML models to other populations may be limited (i.e., commercial insurance, anticoagulated patients, or patients with other cancer types).

## Conclusion

Our study demonstrated a promising application of ML in stroke prediction among older adults with cancer who are newly diagnosed with AFib in the US. This tool may be leveraged in assisting clinicians in identification of patients at high risk of stroke and improving treatment decisions.

### Supplementary Information

Below is the link to the electronic supplementary material.

Supplementary file1 (DOCX 6366 KB)

## Data Availability

The data that support the findings of this study are available on request from the corresponding author. The data are not publicly available due to privacy or ethical restrictions.
