# Supplementary material for: Development and Validation of Machine Learning Algorithms to Predict 1-Year Ischemic Stroke and Bleeding Events in Patients with Atrial Fibrillation and Cancer
Source: Cardiovasc Toxicol. 2024 Mar 18;24(4):365–74. doi: 10.1007/s12012-024-09843-8 (PMC10998799; doi:10.1007/s12012-024-09843-8)
Supplement: Supplementary file 1 — Supplementary file1 (DOCX 6366 KB) [file 12012_2024_9843_MOESM1_ESM.docx]

**Development and validation of machine learning algorithms to predict 1-year ischemic stroke and bleeding in patients with atrial fibrillation and cancer**

Bang Truong, PhD; Jingyi Zheng, PhD; Lori Hornsby, PharmD^,^ Brent Fox, PharmD, PhD; Chiahung Chou, PhD; Jingjing Qian, PhD

Figure S1. Visualization of study design

Figure S2. Summary of approach

Figure S3. Feature importance plot of elastic net algorithm for ischemic stroke prediction (original data)

Figure S4. Feature importance plot of XGBoost algorithm for ischemic stroke prediction (original data)

Figure S5. Feature importance plot of support vector machine algorithm for ischemic stroke prediction (original data)

Figure S6. Feature importance plot of neural network algorithm for ischemic stroke prediction (original data)

Figure S7. Feature importance plot of elastic net algorithm for major bleeding prediction (original data)

Figure S8. Feature importance plot of XGBoost algorithm for major bleeding prediction (original data)

Figure S9. Feature importance plot of support vector machine algorithm for major bleeding prediction (original data)

Figure S10. Feature importance plot of neural network algorithm for major bleeding prediction (original data)

Figure S11. Feature importance plot of elastic net algorithm for ischemic stroke prediction (SMOTE resampled data)

Figure S12. Feature importance plot of elastic net algorithm for ischemic stroke prediction (SMOTE resampled data)

Figure S13. Feature importance plot of XGBoost algorithm for ischemic stroke prediction (SMOTE resampled data)

Figure S14. Feature importance plot of support vector machine algorithm for ischemic stroke prediction (SMOTE resampled data)

Figure S15. Feature importance plot of neural network algorithm for ischemic stroke prediction (SMOTE resampled data)

Figure S16. Feature importance plot of elastic net algorithm for major bleeding prediction (SMOTE resampled data)

Figure S17. Feature importance plot of elastic net algorithm for major bleeding prediction (SMOTE resampled data)

Figure S18. Feature importance plot of XGBoost algorithm for major bleeding prediction (SMOTE resampled data)

Figure S19. Feature importance plot of support vector machine algorithm for major bleeding prediction (SMOTE resampled data)

Figure S20. Feature importance plot of neural network algorithm for major bleeding prediction (SMOTE resampled data)

Table S1. Algorithms to identify study components from SEER-Medicare data

Table S2. Characteristics of patients with new onset AFib and history of cancer in SEER-Medicare registry from 2012 to 2018 (Stroke)

Table S3. Characteristics of patients with new onset AFib and history of cancer in SEER-Medicare registry from 2012 to 2018 (Bleeding)


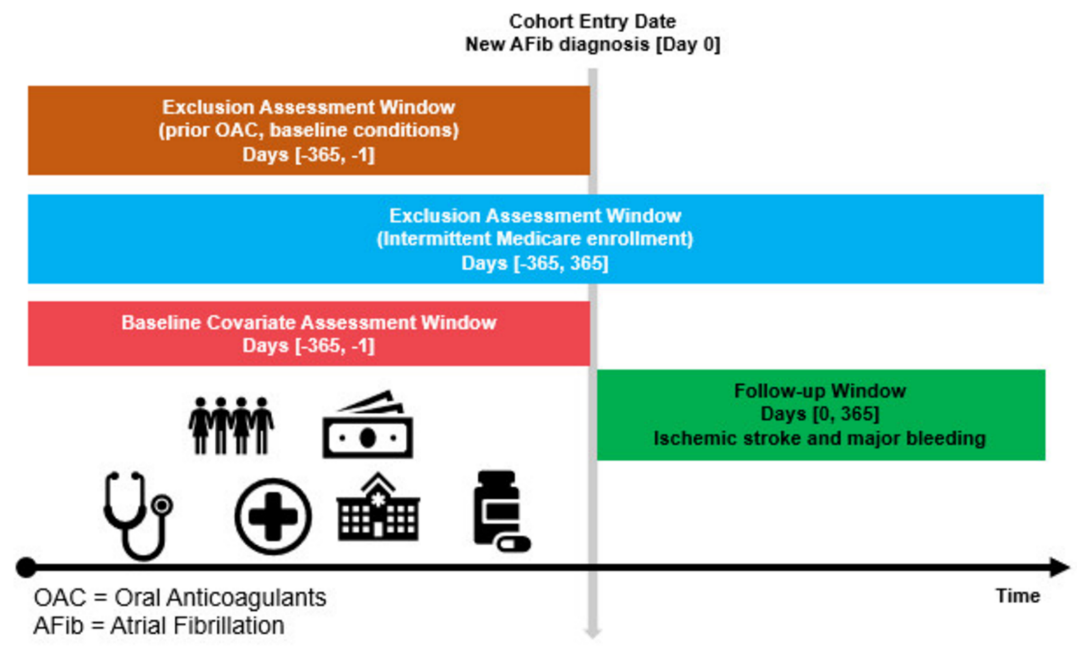


**Figure S1.** Visualization of study design


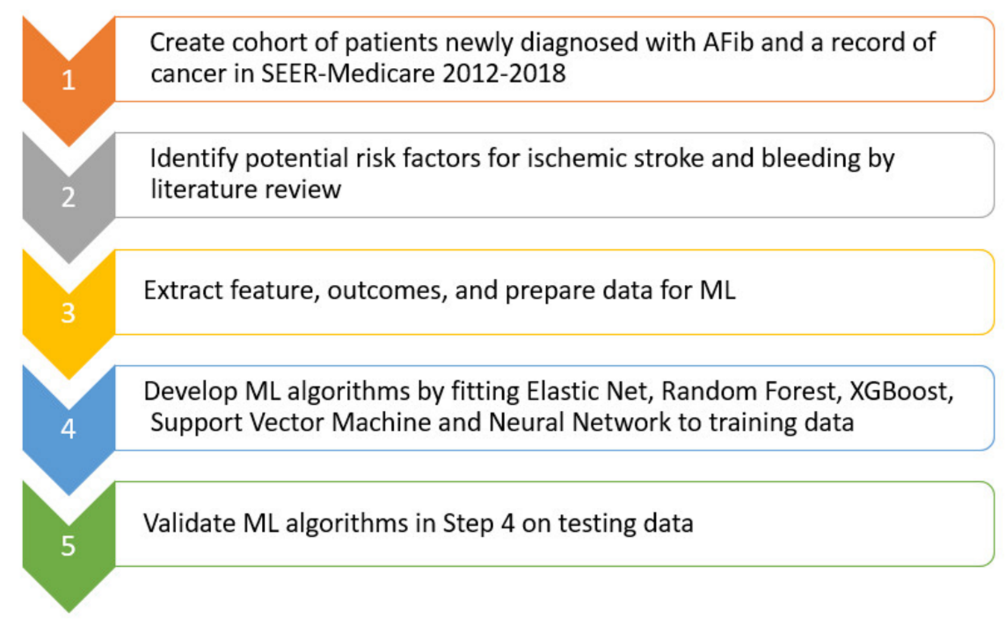


**Figure S2**. Summary of approach


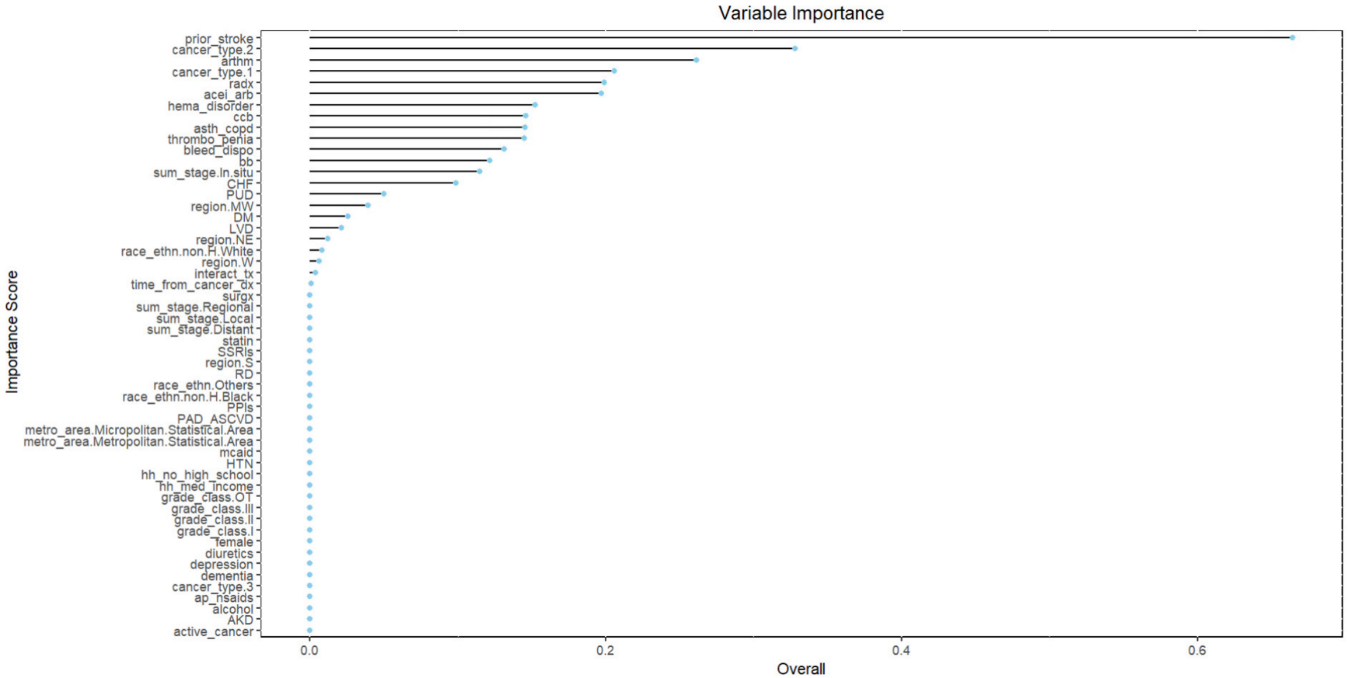


**Figure S3**. Feature importance plot of elastic net algorithm for ischemic stroke prediction (original data)


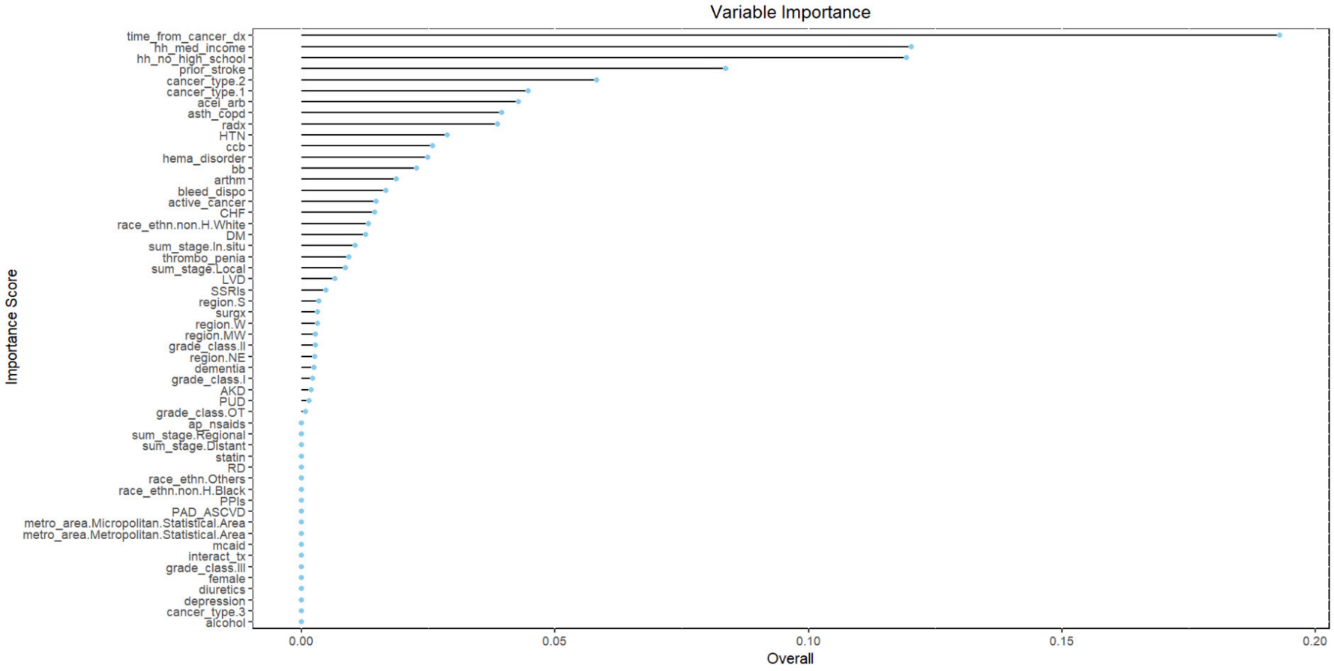


**Figure S4**. Feature importance plot of XGBoost algorithm for ischemic stroke prediction (original data)


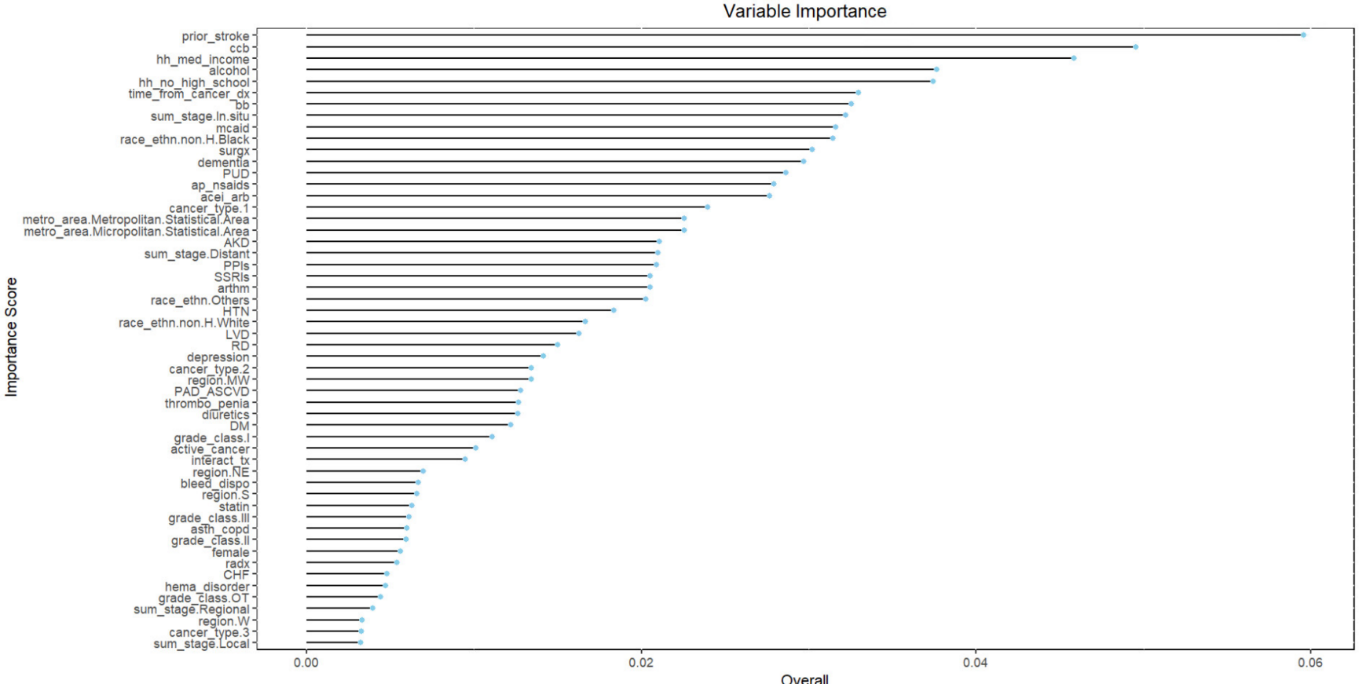


**Figure S5**. Feature importance plot of support vector machine algorithm for ischemic stroke prediction (original data)


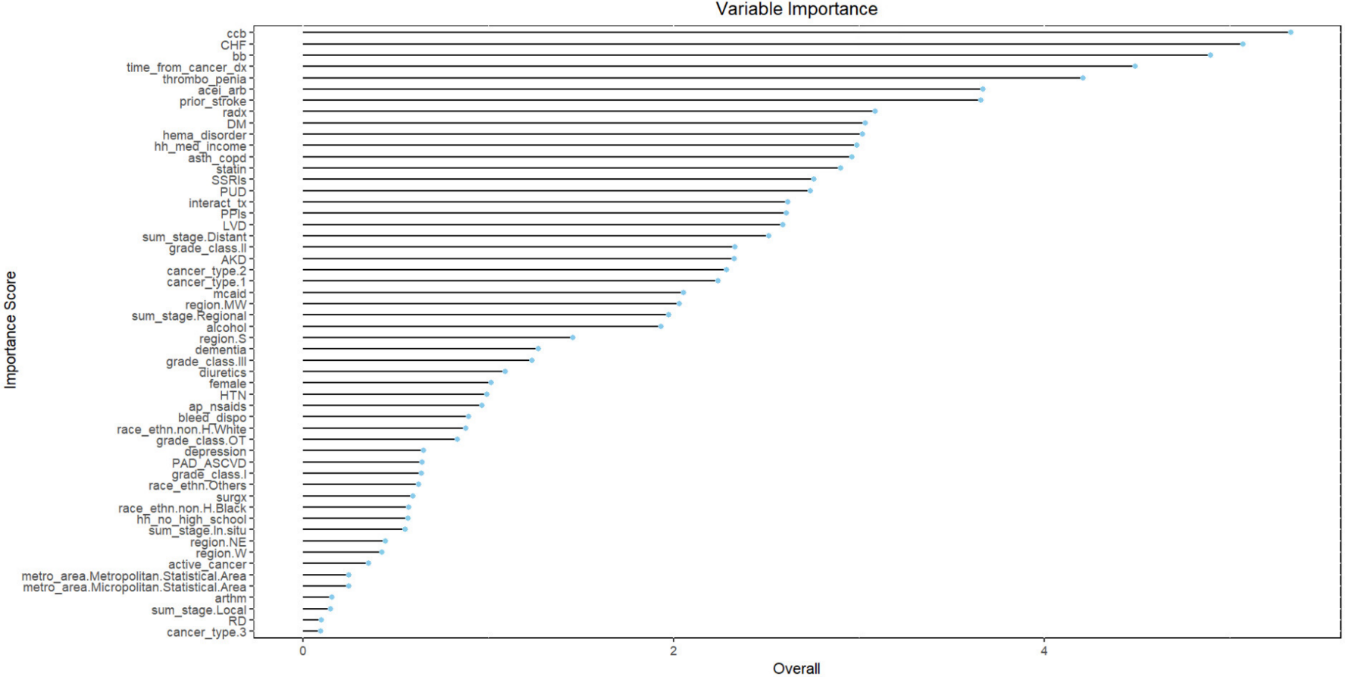


**Figure S6**. Feature importance plot of neural network algorithm for ischemic stroke prediction (original data)


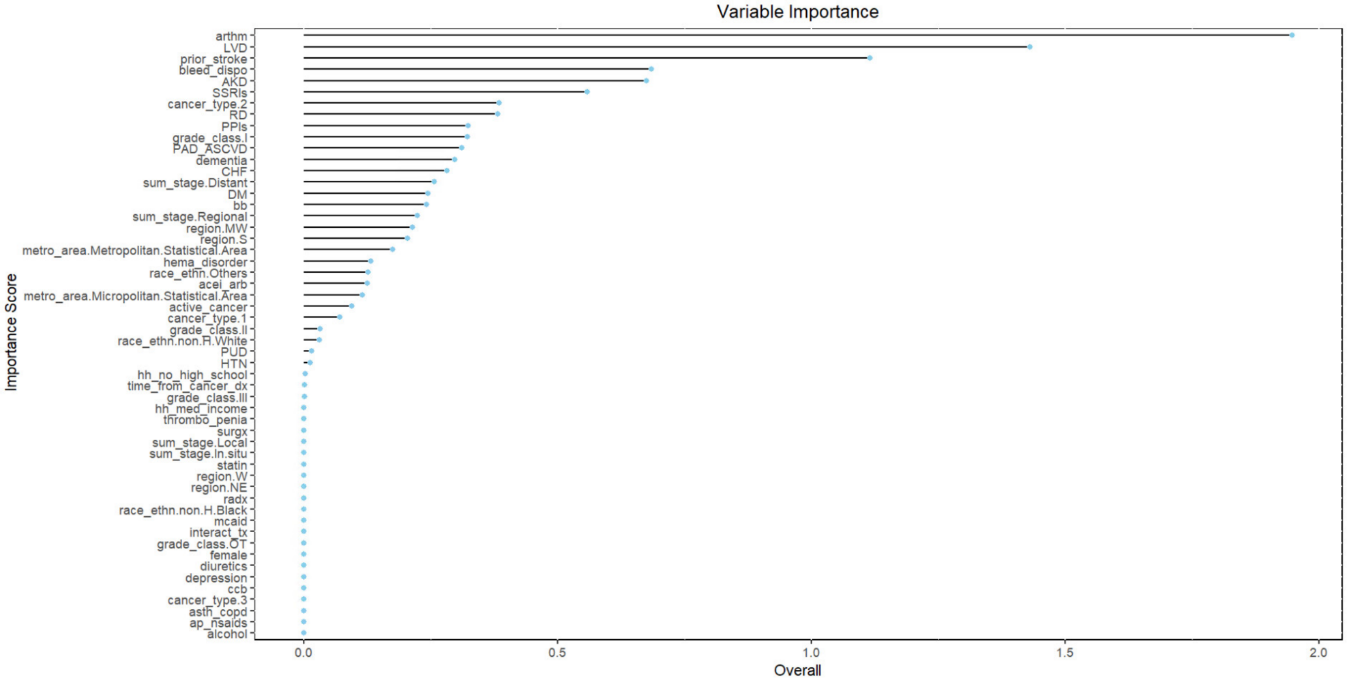


**Figure S7**. Feature importance plot of elastic net algorithm for major bleeding prediction (original data)


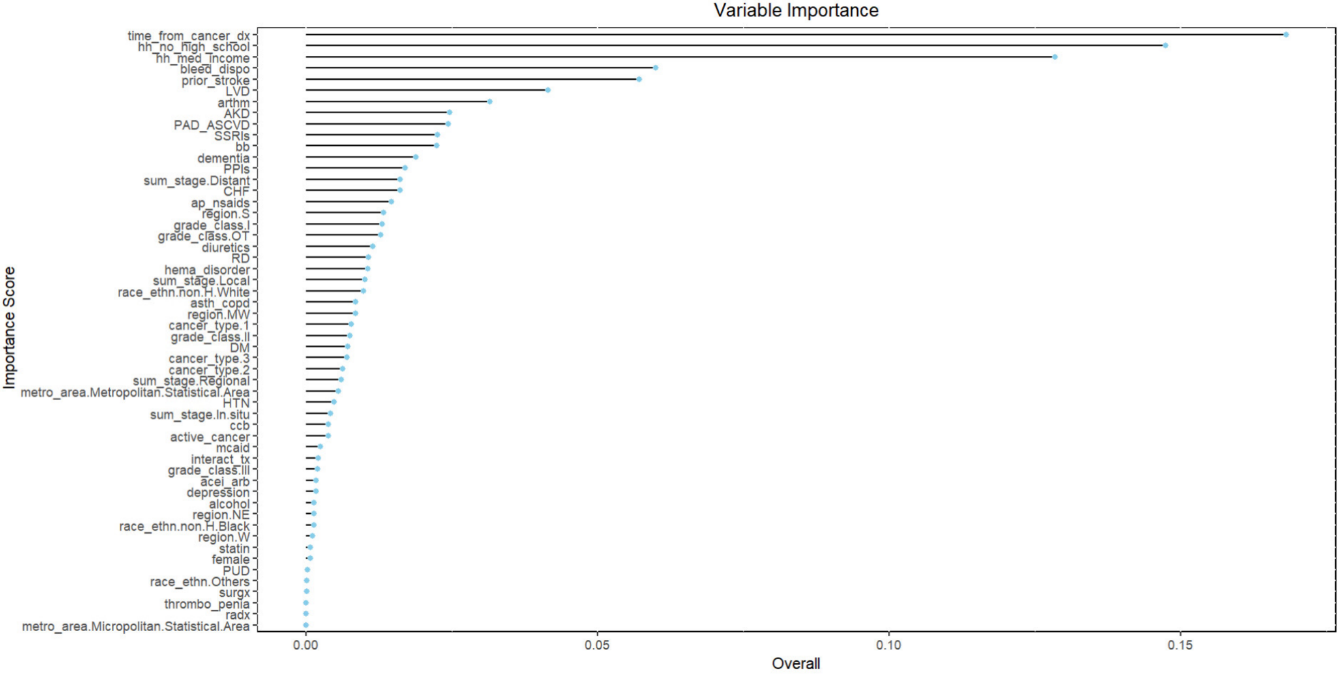


**Figure S8**. Feature importance plot of XGBoost algorithm for major bleeding prediction (original data)


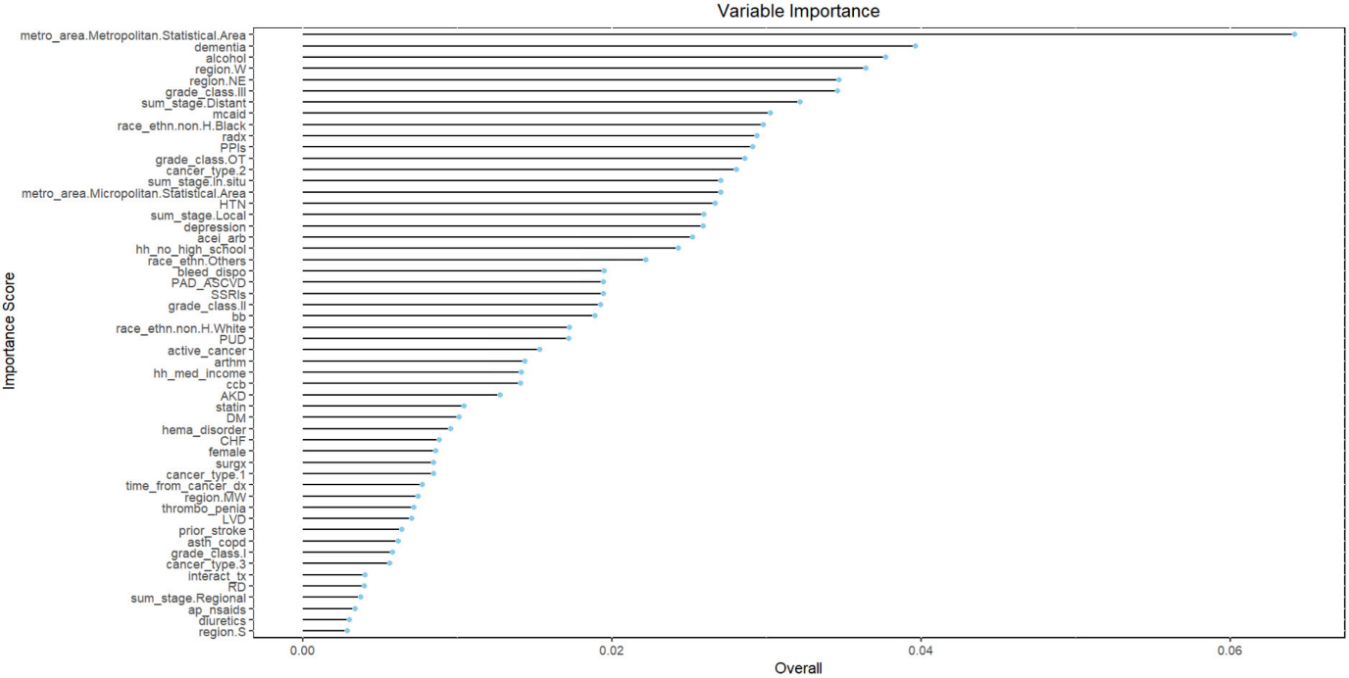


**Figure S9.** Feature importance plot of support vector machine algorithm for major bleeding prediction (original data)


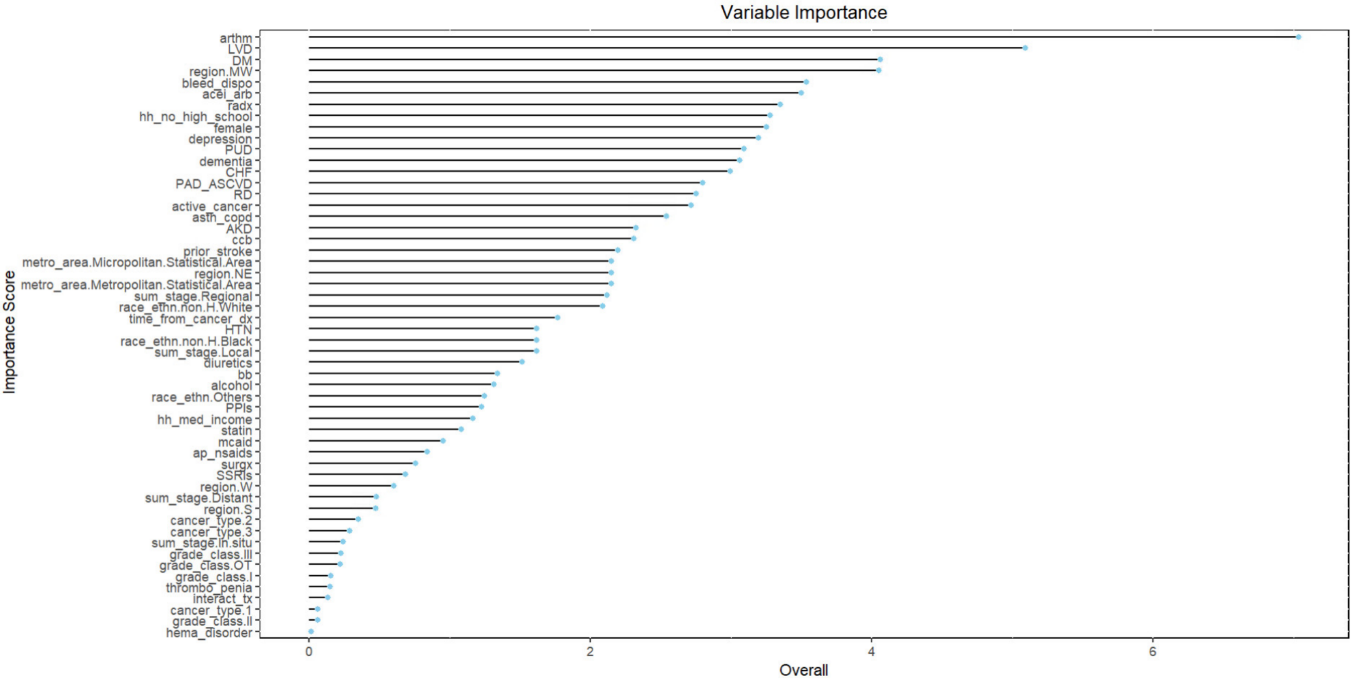


**Figure S10**. Feature importance plot of neural network algorithm for major bleeding prediction (original data)


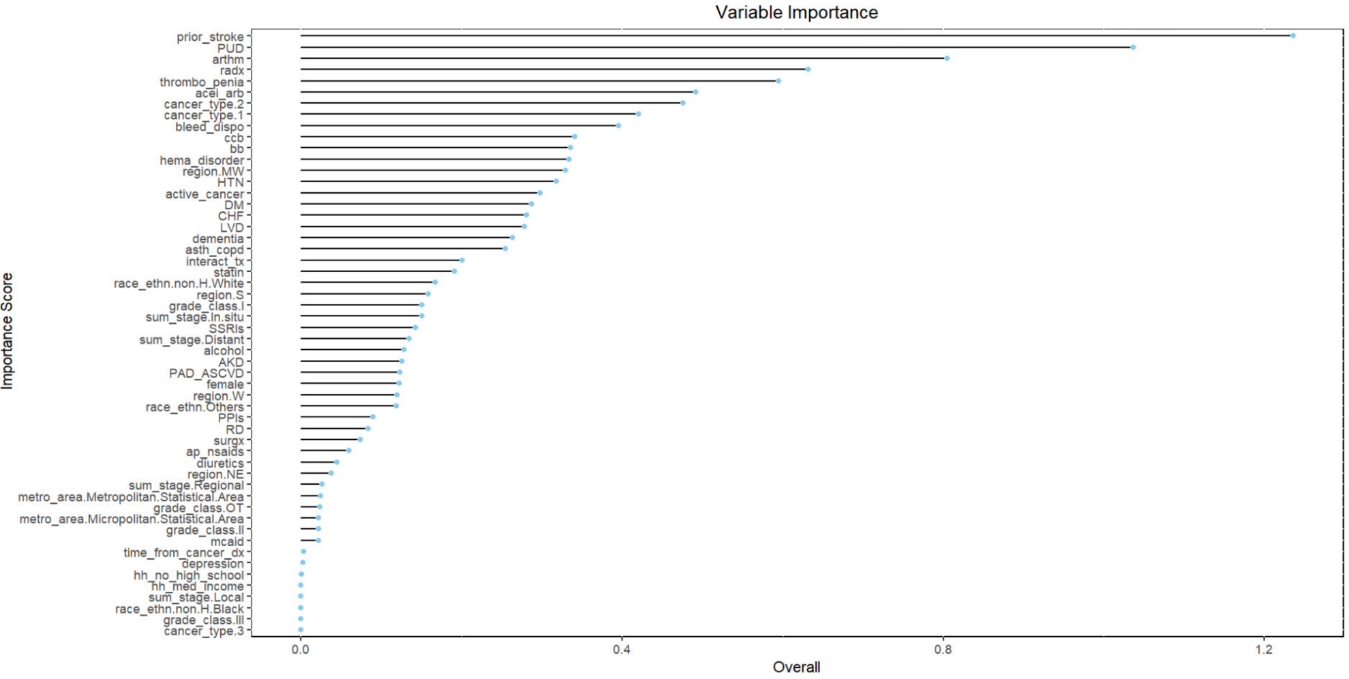


**Figure S11**. Feature importance plot of elastic net algorithm for ischemic stroke prediction (SMOTE resampled data)


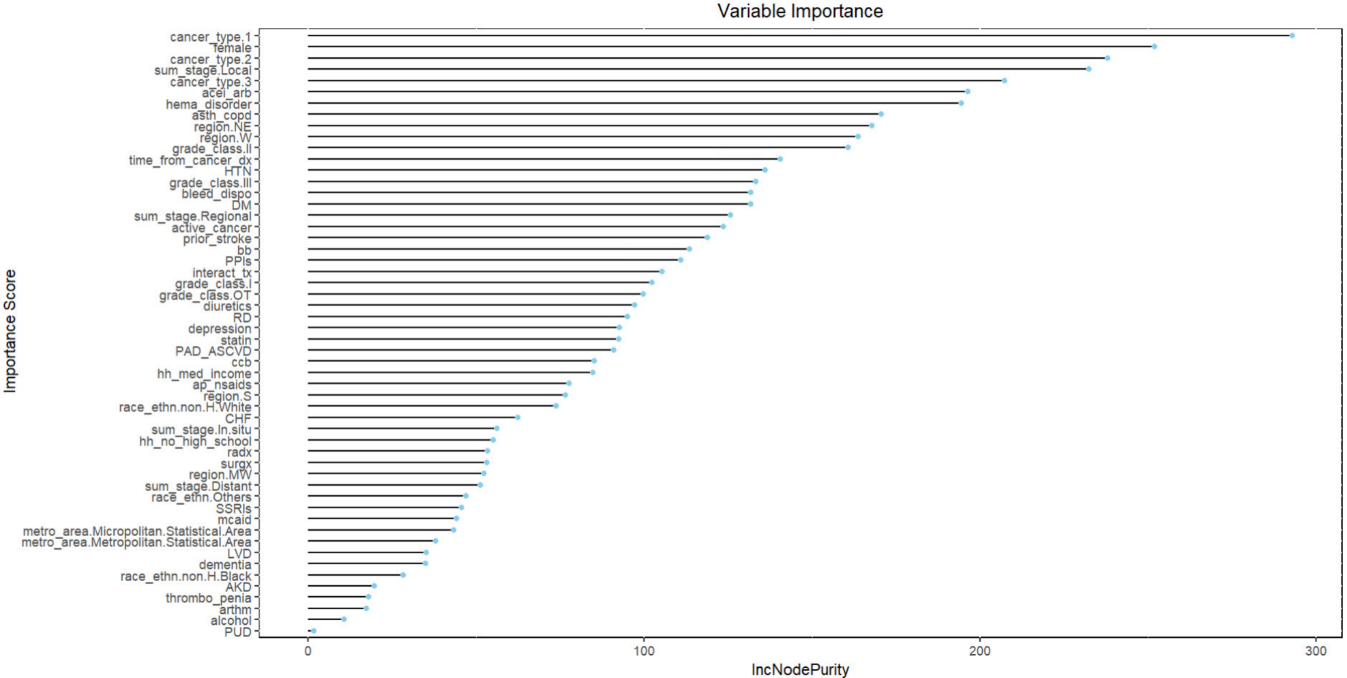


**Figure S12**. Feature importance plot of elastic net algorithm for ischemic stroke prediction (SMOTE resampled data)


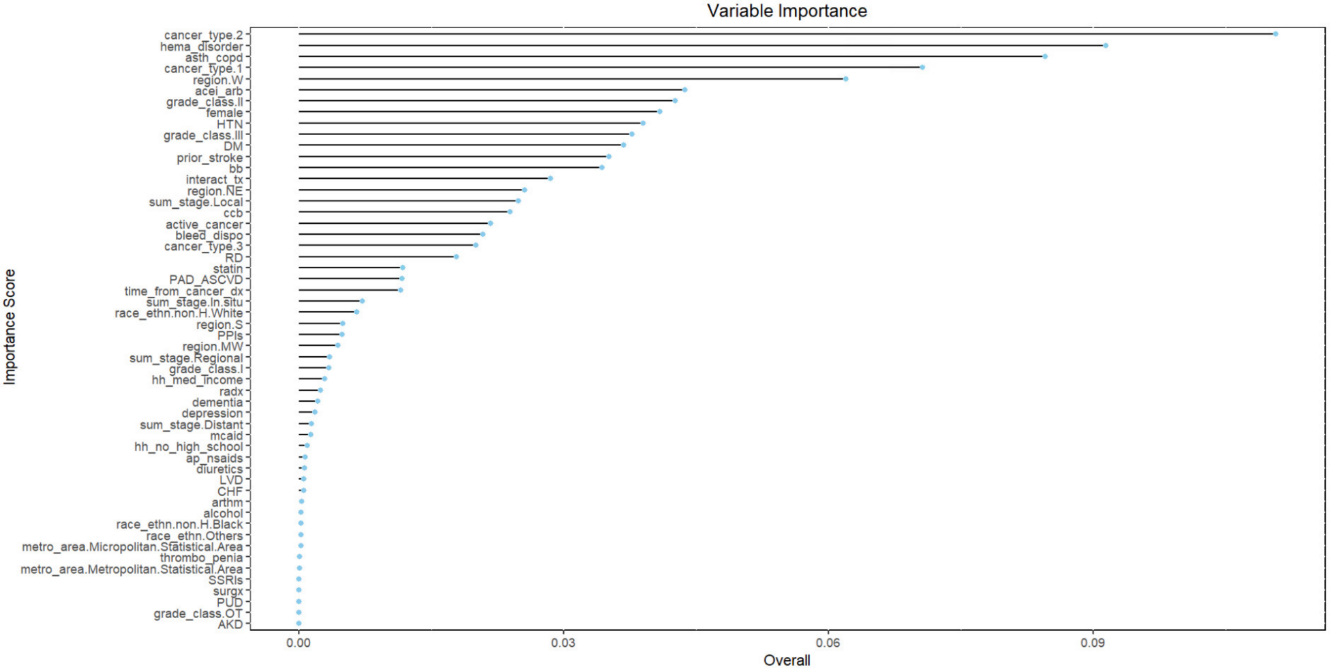


**Figure S13**. Feature importance plot of XGBoost algorithm for ischemic stroke prediction (SMOTE resampled data)


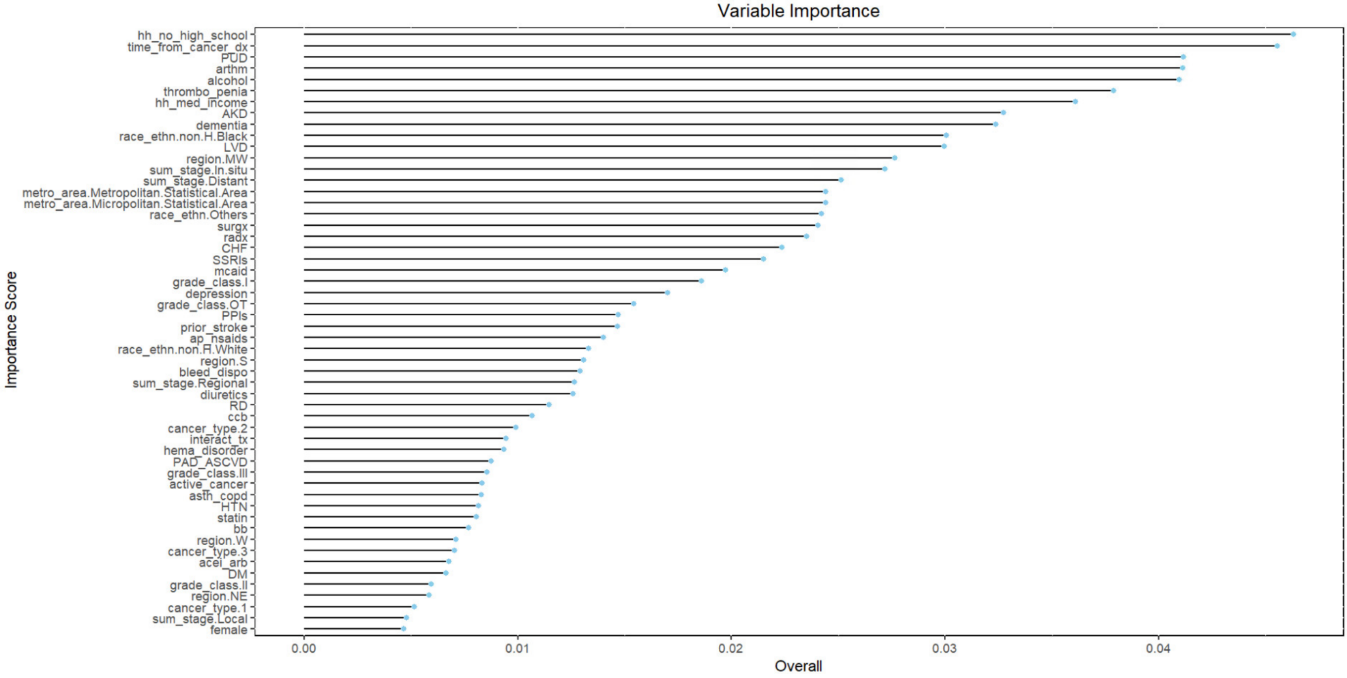


**Figure S14**. Feature importance plot of support vector machine algorithm for ischemic stroke prediction (SMOTE resampled data)


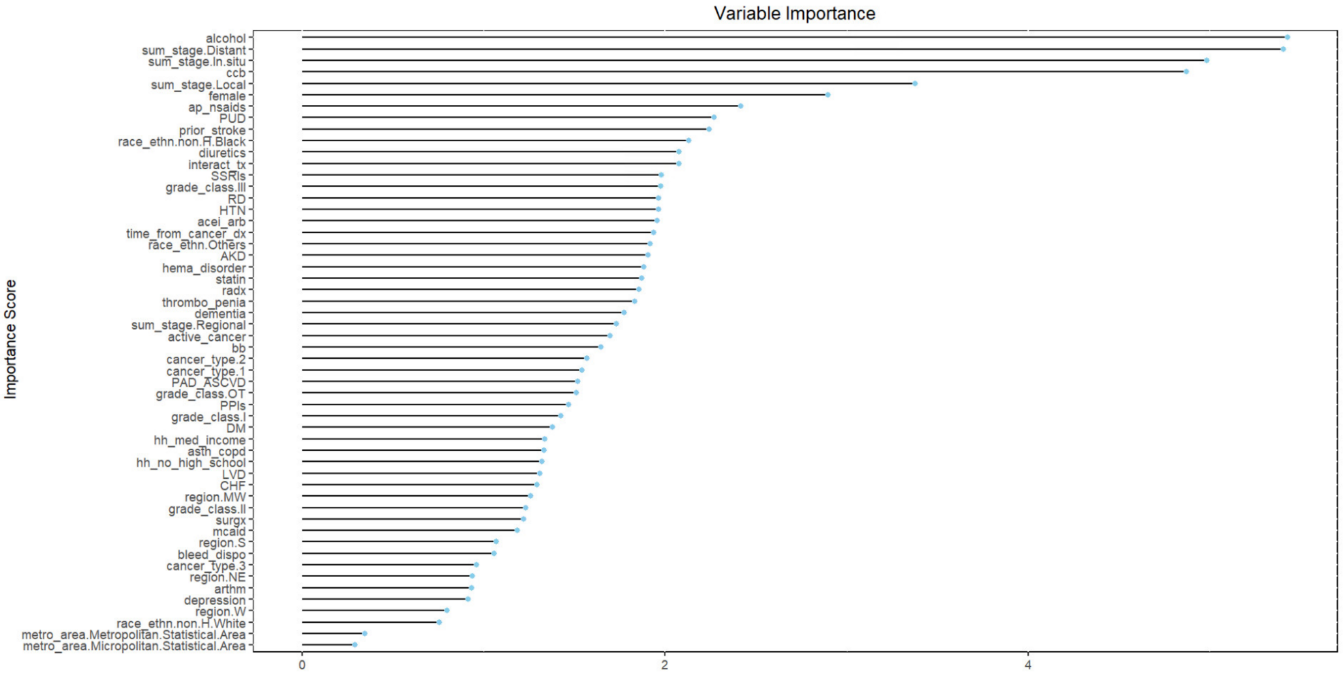


**Figure S15**. Feature importance plot of neural network algorithm for ischemic stroke prediction (SMOTE resampled data)


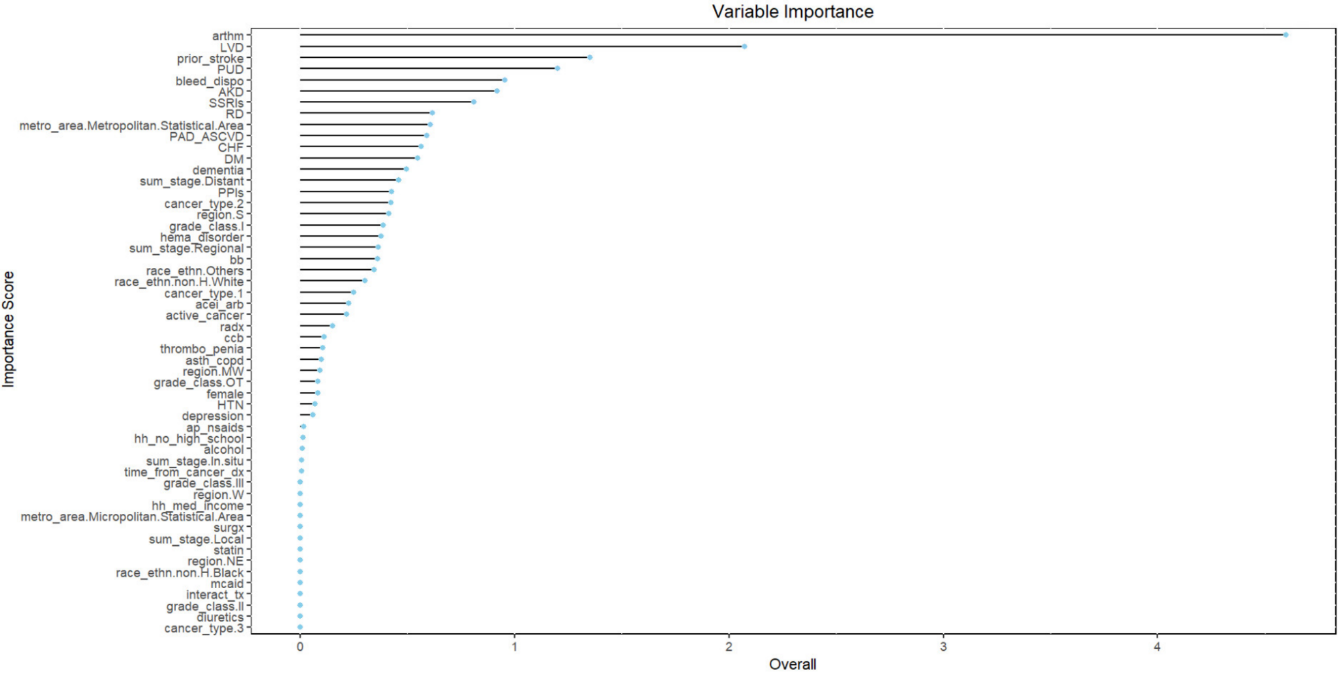


**Figure S16**. Feature importance plot of elastic net algorithm for major bleeding prediction (SMOTE resampled data)


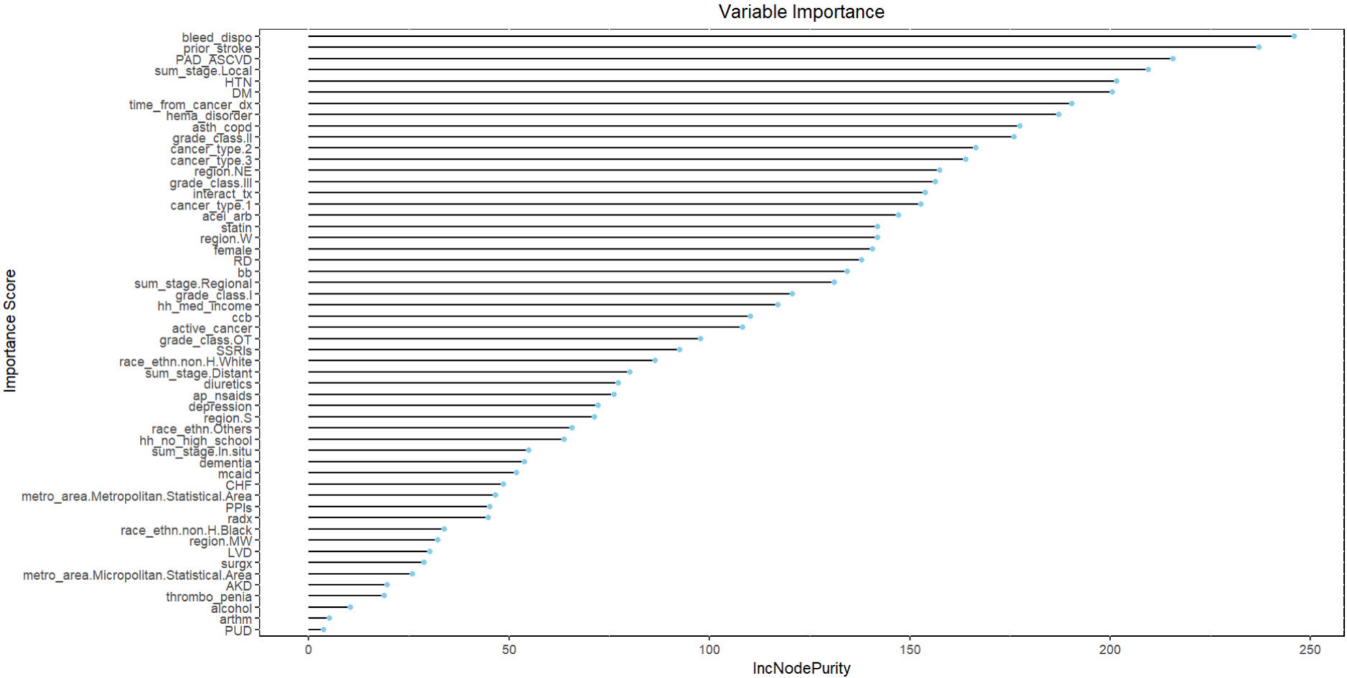


**Figure S17**. Feature importance plot of elastic net algorithm for major bleeding prediction (SMOTE resampled data)


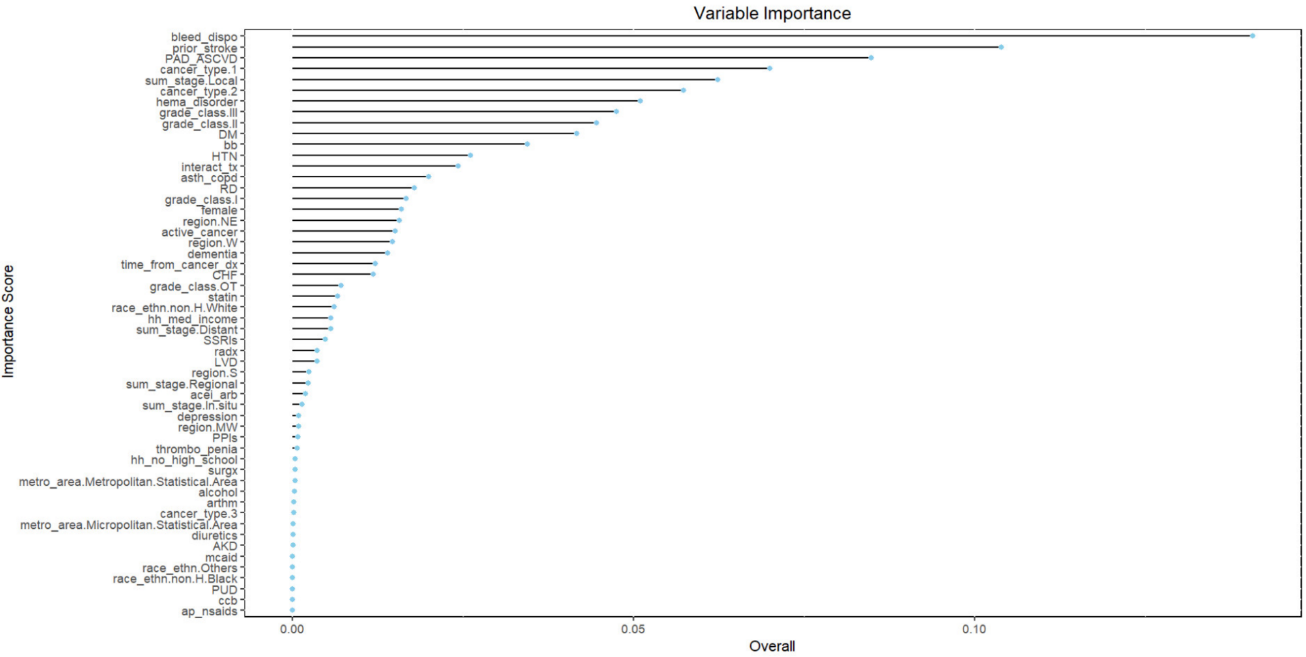


**Figure S18**. Feature importance plot of XGBoost algorithm for major bleeding prediction (SMOTE resampled data)


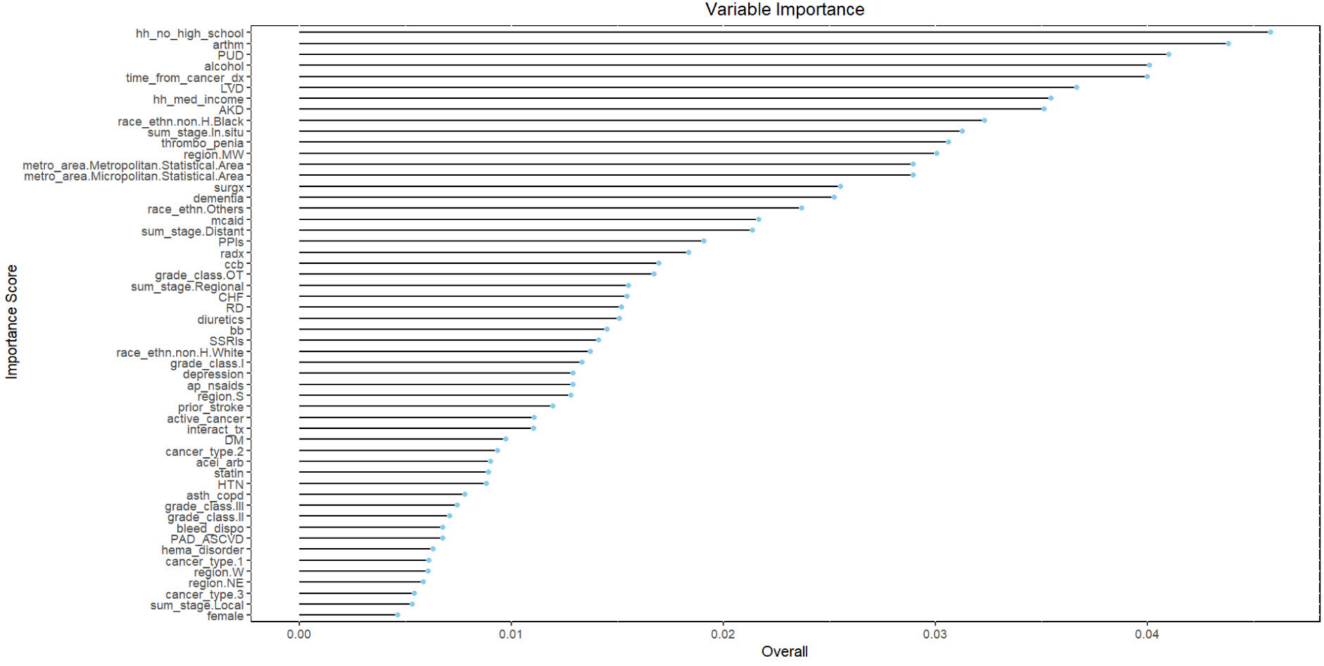


**Figure S19**. Feature importance plot of support vector machine algorithm for major bleeding prediction (SMOTE resampled data)


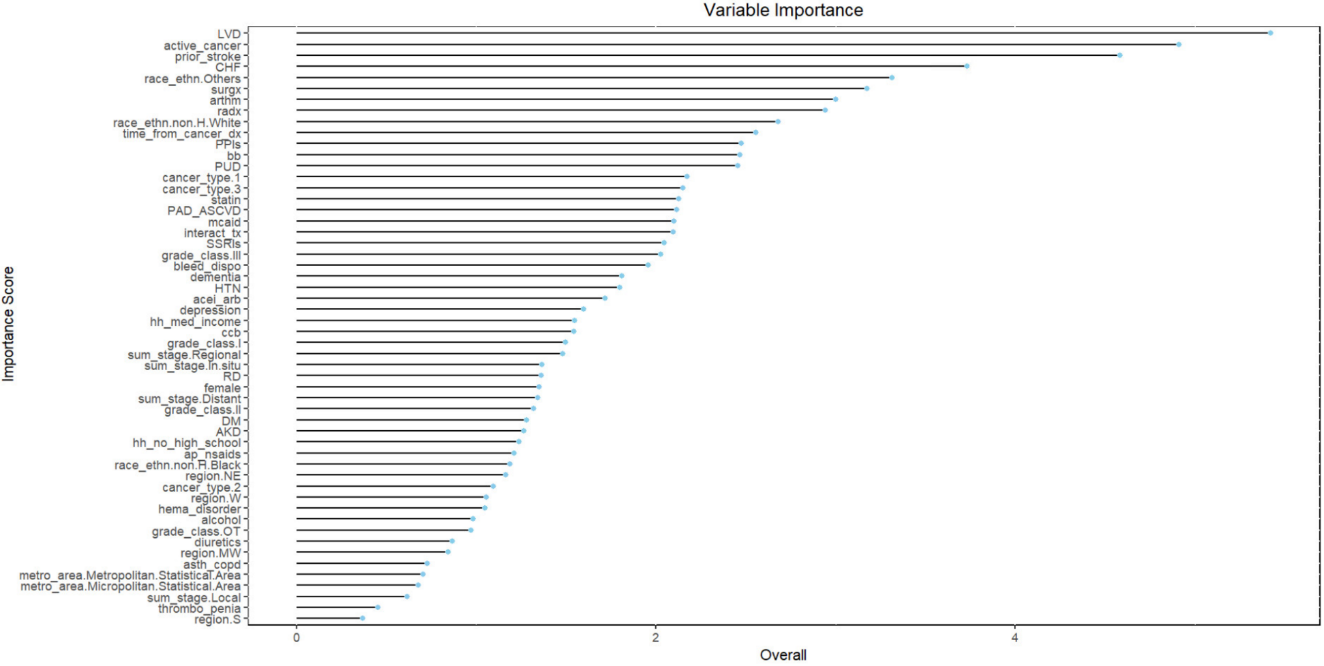


**Figure S20**. Feature importance plot of neural network algorithm for major bleeding prediction (SMOTE resampled data)

**Table S1.** Algorithms to identify study components from SEER-Medicare data

| **Component** | **Code type** | **Codes** |
| --- | --- | --- |
| ***Eligibility criteria*** | | |
| AFib | ICD-9-CM | 427.31 or 427.32 |
|  | ICD-10-CM | I48.xx |
| Breast cancer | ICD-O-3 | C50.0-C50.9 |
| Lung cancer | ICD-O-3 | C34.0, C34.1, C34.2, C34.3, C34.8, C34.9, C33.9 |
| Prostate cancer | ICD-O-3 | C61.9 |
| Exclusion criteria |  |  |
| Valvular heart diseases | ICD-9-CM | 0932, 394, 395, 396, 3970, 3971, 240, 4241, 242, 4243, 7460, 7461, 7462, 7463, 7464, 7465, 7466, 99602, 99671, V422 |
|  | ICD-10-CM | I05, I06, I07, I08, I34, I35, I36, I37, I39, Q22, Q23, T820, T8201, T8202, T8203, T8209, T8222, T826, Z952, Z953, Z954 |
|  | ICD-9-CM PX | 351, 352, 3533, 3595, 3599 |
|  | ICD-10-PCS | 02RF, 02RG, 02RH, 02RJ, 02QF, 02QG, 02QH, 02QJ |
| Heart valve repair or replacement | ICD-9-CM | V433 |
| VTE | ICD-9-CM | 4151 ,453, V1251, V1255 |
|  | ICD-10-CM | I26, I80, I81, I82, Z8671 |
| Joint replacement | ICD-9-CM PX | 8151, 8152, 8154 |
|  | ICD-10-PCS | 0SR9019, 0SR901A, 0SR901Z, 0SR9029, 0SR902A, 0SR902Z, 0SR9039, 0SR903A, 0SR903Z, 0SR9049, 0SR904A, 0SR904Z, 0SR9069, 0SR906A, 0SR906Z, 0SR907Z, 0SR90EZ, 0SR90J9, 0SR90JA, 0SR90JZ, 0SR90KZ, 0SRA009, 0SRA00A, 0SRA00Z, 0SRA019, 0SRA01A, 0SRA01Z, 0SRA039, 0SRA03A, 0SRA03Z, 0SRA07Z, 0SRA0J9, 0SRA0JA, 0SRA0JZ, 0SRA0KZ, 0SRB019, 0SRB01A, 0SRB01Z, 0SRB029, 0SRB02A, 0SRB02Z, 0SRB039, 0SRB03A, 0SRB03Z, 0SRB049, 0SRB04A, 0SRB04Z, 0SRB069, 0SRB06A, 0SRB06Z, 0SRB07Z, 0SRB0EZ, 0SRB0J9, 0SRB0JZ, 0SRB0KZ, 0SRE009, 0SRE00A, 0SRE00Z, 0SRE019, 0SRE01A, 0SRE039, 0SRE03A, 0SRE03Z, 0SRE07Z, 0SRE0J9, 0SRE0JA, 0SRE0JZ, 0SRR019, 0SRR01A, 0SRR01Z, 0SRR039, 0SRR03A, 0SRR03Z, 0SRB0JA, 0SRR07Z, 0SRR0J9, 0SRR0JA, 0SRR0JZ, 0SRR0KZ, 0SRS019, 0SRE01Z, 0SRS01A, 0SRS01Z, 0SRS039, 0SRS03A, 0SRS03Z, 0SRS07Z, 0SRE0KZ, 0SRS0J9, 0SRS0JA, 0SRS0JZ, 0SRS0KZ, 0SRC069, 0SRC06A, 0SRC06Z, 0SRC07Z, 0SRC0EZ, 0SRC0J9, 0SRC0JA, 0SRC0JZ, 0SRC0KZ, 0SRC0L9, 0SRC0LA, 0SRC0LZ, 0SRC0M9, 0SRC0MA, 0SRC0MZ, 0SRC0N9, 0SRC0NA, 0SRC0NZ, 0SRD069, 0SRD06A, 0SRD06Z, 0SRD07Z, 0SRD0EZ, 0SRD0J9, 0SRD0JA, 0SRD0JZ, 0SRD0KZ, 0SRD0L9, 0SRD0LA, 0SRD0LZ, 0SRD0M9, 0SRD0MA, 0SRD0MZ, 0SRD0N9, 0SRD0NA, 0SRD0NZ, 0SRT07Z, 0SRT0J9, 0SRT0JA, 0SRT0JZ, 0SRT0KZ, 0SRU07Z, 0SRU0J9, 0SRU0JA, 0SRU0JZ, 0SRU0KZ, 0SRV07Z, 0SRV0J9, 0SRV0JA, 0SRV0JZ, 0SRV0KZ, 0SRW07Z, 0SRW0J9, 0SRW0JA, 0SRW0JZ, 0SRW0KZ |
| Renal impairment stage 5/ESRD | ICD-9-CM | 40301, 40311, 40391, 5855, 5856, V451, V56 |
|  | ICD-10-CM | I120, I1311, I132, N185, Y841, Z49, Z9115, Z992 |
|  | ICD-9-CM PX | 3995, 5498 |
|  | ICD-10-PCS | 3E1M39Z, 5A1D70Z, 5A1D80Z, 5A1D90Z |
| History of stroke/TIA | ICD-9-CM | 36231, 36232, 36233, 36234, 43301, 43311, 43321, 43331, 43381, 43391, 43401, 43411, 436, 430, 431, 43391, 435 |
|  | ICD-10-CM | H340, H341, H342, I63, I60, I61, I62, I63, G45, I6782, I6789 |
| Major surgery |  | Not included due to large number of codes |
| Intracranial/Spinal bleeding | ICD-9-CM | 430, 431, 432, 852, 853, |
|  | ICD-10-CM | I60, I61, I62, S064, S065, S066 |
| Intraocular bleeding | ICD-9-CM | 36281, 37923, 36361, 36362, |
|  | ICD-10-CM | H356, H3130, H3131, H431 |
| Retroperitoneal bleeding | ICD-9-CM | 56881 |
|  | ICD-10-CM | K661 |
| Atraumatic intra-articular bleeding | ICD-9-CM | 7191 |
|  | ICD-10-CM | M250 |
| Gastrointestinal bleeding | ICD-9-CM | 4560, 45620, 5301, 5307, 53082, 5310, 5311, 5312, 5313, 5314, 5315, 5316, 5317, 5319, 5320, 5321, 5322, 5323, 5324, 5325, 5326, 5327, 5329, 5330, 5331, 5332, 5333, 5334, 5335, 5336, 5337, 5339, 5340, 53400, 53401, 5341, 5342, 5343, 5344, 5345, 5346, 5347, 5349, 53500, 53501, 53510, 53511, 53520, 53521, 53530, 53531, 53540, 53541, 53550, 53551, 53560, 53561, 53783, 5780, 4551, 4552, 4554, 4555, 4556, 4557, 4558, 4559, 56200, 56201, 56202, 56203, 56210, 56211, 56212, 56213, 5693, 56985, 5781, 5789 |
|  | ICD-10-CM | I8501, I8511, K20, K210, K2211, K226, K250, K251, K252, K254, K255, K256, K260, K261, K262, K264, K265, K266, K270, K271, K272, K274, K275, K276, K280, K281, K282, K284, K285, K286, K2901, K2921, K2931, K2941, K2951, K2961, K2971, K2981, K2991, K31811, K920, K5521, K5701, K5711, K5721, K5731, K5741, K5751, K5753, K5781, K5791, K5793, K625, K640, K641, K642, K643, K644, K645, K648, K649, K921, K922 |
| ***Outcomes*** | | |
| Ischemic stroke (new diagnosis) | ICD-9-CM | 36231, 36232, 36233, 36234, 43301, 43311, 43321, 43331, 43381, 43391, 43401, 43411, 436 |
|  | ICD-10-CM | H340, H341, H342, I63 |
| Major bleeding | ICD-9-CM | 3361, 36361, 36362, 36372, 37632, 37742, 37923, 4230, 430, 431, 432, 56881, 7191, 72992, 852, 853, 86601, 86602, 86611, 86612 |
|  | ICD-10-CM | G9519, H0523, H3130, H3131, H3141, H431, H4702, I230, I312, I60, I61, I62, K661, M250, M7981, S064, S065, S066, S260, S3701, S3702, S3703, S3704, S3705, S3706 |
| VTE | ICD-9-CM | See eligibility criteria |
|  | ICD-10-CM |  |
| Intracranial bleeding | ICD-9-CM | See eligibility criteria |
|  | ICD-10-CM |  |
| GI bleeding | ICD-9-CM | See eligibility criteria |
|  | ICD-10-CM |  |
| Non-critical site bleeding | ICD-9-CM | 2800, 2851, 4551, 4552, 4554, 4555, 4556, 4557, 4558, 4559, 4560, 45620, 4590, 5301, 5307, 5310, 5311, 5312, 5313, 5314, 5315, 5316, 5317, 5319, 5320, 5321, 5322, 5323, 5324, 5325, 5326, 5327, 5329, 5330, 5331, 5332, 5333, 5334, 5335, 5336, 5337, 5339, 5340, 5341, 5342, 5343, 5344, 5345, 5346, 5347, 5349, 53500, 53501, 53510, 53511, 53520, 53521, 53530, 53531, 53540, 53541, 53550, 53551, 53560, 53561, 53783, 56200, 56201, 56202, 56203, 56210, 56211, 56212, 56213, 5693, 56985, 5780, 5781, 5789, 59381, 59970, 59971, 6236, 6238, 6266, 6268, 7847, 7848, 7863, D500, D62, I8501, I8511, K20, K210, K2211, K226, K250 |
|  | ICD-10-CM | K251, K252, K254, K255, K256, K260, K261, K262, K264, K265, K266, K270, K271, K272, K274, K275, K276, K280, K281, K282, K284, K285, K286, K2901, K2921, K2931, K2941, K2951, K2961, K2971, K2981, K2991, K31811, K5521, K5701, K5711, K5721, K5731, K5741, K5751, K5753, K5781, K5791, K5793, K625, K640, K641, K642, K643, K644, K645, K648, K649, K920, K921, K922, N897, N898, N921, N938, N939, R040, R041, R042, R310, R319, R58 |
| ***Covariates*** | | |
| CHF | ICD-9-CM | 39891, 40201, 40211, 40291, 40401, 40403, 40411, 40413, 40491, 40493, 4254, 4259, 428 |
|  | ICD-10-CM | I0981, I110, I130, I132, I425, I428, I50 |
| HTN | ICD-9-CM | 401, 402, 403, 404, 405 |
|  | ICD-10-CM | I10, I11, I12, I13, I14, I15, I16 |
| DM | ICD-9-CM | 250, 3572, 3620, 36641, |
|  | ICD-10-CM | E10, E11, E13 |
| Vascular diseases | ICD-9-CM | 410, 412, 4400, 4402, 4403, 4409, 4442, 4439, 44481 |
|  | ICD-10-CM | I21, I252, I700, I702, I703, I704, I705, I706, I707, I709, I742, I743, I744, I739, I745 |
| Renal diseases | ICD-9-CM | 0160, 0954, 1890, 1899, 2230, 23691, 2504, 2714, 2741, 28311, 403, 404, 4401, 4421, 4473,  5724, 580, 581, 582, 583, 584, 585, 586, 587, 588, 591, 6421, 6462, 75312, 75313, 75314, 75315, 75316, 75317, 75319, 7532, 7944, V420, V451, V56 |
|  | ICD-10-CM | A1811, A5275, C649, C689, D4100, E1129, E1029, E1121, E1021, E748, M1030, N200, D593, I120, I129, I1310, I130, I1311, I132, I1311, I701, I722, I773, K767, N00, N01, N02, N03, N04, N05, N06, N07, N08, N1330, O10419, O10411, O10412, O10413, O1042, O1043, O26839, O1214, O26831, O26832, O26833, Q613, Q612, Q6119, Q614, Q615,  Q6102, Q618, Q6239, Q6211, Q6212, Q6231, Q6210, Q6211, R944, Z940, Z992, Z9115, Z4931, Z4901, Z4902, Z4932 |
| Liver diseases | ICD-9-CM | 070, 07271, 09162, 1305, 571, 573, 7948 |
|  | ICD-10-CM | A5145, B0081, B15, B16, B17, B18, B19, B251, B2681, B581, B942, K70, K71, K72, K73, K74, K75, K76, K77, R94.5 |
| Bleeding disposition | ICD-9-CM | 430, 431, 432, 56881, 5997, 5307, 5310, 5312, 5314, 5316, 5320, 5322, 5324, 5326, 5330, 5332, 5334, 5336, 5340, 5342, 5344, 5346, 5693, 53501, 53511, 53521, 53531, 53541, 53551, 53561, 53571, 53783, 53784, 56202, 56203, 56212, 56213, 56985, 578, 7847, 7863, 6262, 7191, 37272, 459 |
|  | ICD-10-CM | I60, I61, I62, K661, R31, K226, K250, K252, K254, K256, K260, K262, K264, K266, K270, K272, K274, K276, K280, K282, K284, K286, K625, K2901, K2921, K2931, K2941, K2951, K2961, K2971, K2981, K2991, K31811, K3182, K5701, K5711, K5713, K5721, K5731, K5733, K5741, K5751, K5753, K5781, K5791, K5793, K5521, K920, K921, K922, R040, R042, N920, M250, M122, H113, R58 |
| Alcohol use disorders | ICD-9-CM | 291, 303, 3050, 3575, 4255, 5353, 5710, 5711, 5712, 5713, 7903 |
|  | ICD-10-CM | E8600, V113, F10, Z714 |
| Asthma/COPD | ICD-9-CM | 491, 492, 496, 49300, 49301, 49302, 49310, 49311, 49312, 49320, 49321, 49322, 49381, 49382, 49390, 49391, 49392 |
|  | ICD-10-CM | J41, J42, J43, J44, J4520, J4521, J4522, J4530, J4531, J4532, J4540, J4541, J4542, J4550, J4551, J4552, J45901, J45902, J45909, J45990, J45991, J45998 |
| Hematological disorders | ICD-9-CM | 280, 281, 282, 283, 284, 285, 286, 2871, 2873, 2874, 2875 |
|  | ICD-10-CM | D46, D50, D51, D52, D53, D55, D56, D57, D58, D59, D60, D61, D62, D63, D64 |
| Dementia | ICD-9-CM | 3310, 3311, 3312, 3317, 290, 2940, 2941, 2948, 797 |
|  | ICD-10-CM | G30, G310, G311, G312, G319, F02, F03, F04, R4181 |
| Depression | ICD-9-CM | 2962, 2963, 2965, 3004, 309, 311 |
|  | ICD-10-CM | F32, F33, F341, F43 |
| Thrombocytopenia | ICD-9-CM | 286, 287 |
|  | ICD-10-CM | D68, D69 |
| AKD | ICD-9-CM | 584 |
|  | ICD-10-CM | N17 |
| Peptic ulcer diseases | ICD-9-CM | 533, V1271, |
|  | ICD-10-CM | K27, Z8711 |
| Aspirin/NSAIDs | Generic drug name | ASPIRIN, CLOPIDOGREL, CELECOXIB, DICLOFENAC, DIFLUNISAL, ETODOLAC, FENOPROFEN, FLURBUPROFEN, IBUPROFEN, INDOMETHACIN, KETOPROFEN, KETOROLAC, MEFENAMIC, MELOXICAM, NABUMETONE, NAPROXEN, OXAPROZIN, PIROXICAM, SULINDAC, TOLMETIN |
| ACEI/ARB | Generic drug name | BENAZEPRIL, CAPTOPRIL, ENALAPRIL, FOSINOPRIL, LISINOPRIL, MOEXIPRIL, PERINDOPRIL, QUINAPRIL, RAMIPRIL, TRANDOLAPRIL LOSARTAN, IRBESARTAN, OLMESARTAN, VALSARTAN, TELMISARTAN, CANDESARTAN, AZILSARTAN |
| CCB | Generic drug name | AMLODIPINE, DILTIAZEM, FELODIPINE, ISRADIPINE, LEVAMLODIPINE, NIFEDIPINE, NISOLDIPINE, VERAPAMIL |
| BB | Generic drug name | ACEBUTOLOL, ATENOLOL, BETAXOLOL, BISOPROLOL, CARVEDILOL, LABETALOL, METOPROLOL , NADOLOL, NEBIVOLOL, PINDOLOL, PROPRANOLOL, TIMOLOL |
| Antiarrhythmic drugs | Generic drug name | QUINIDINE, PROCAINAMIDE, DISOPYRAMIDE, LIDOCAINE , MEXILETINE, FLECAINIDE, PROPAFENONE, AMIODARON, EDRONEDARONE, DOFETILIDE, SOTALOL, IBUTILID, DIGOXIN |
| Diuretics | Generic drug name | HYDROCHLOROTHIAZIDE, CHLOROTHIAZIDE, CHLORTHALIDONE, EPLERENONE, FUROSEMIDE, INDAPAMIDE, SPIRONOLACTONE , TORSEMIDE, METOLAZONE |
| Statins | Generic drug name | ATORVASTATIN, FLUVASTATIN, LOVASTATIN, PITAVASTATIN, PRAVASTATIN, ROSUVASTATIN, SIMVASTATIN |
| PPIs | Generic drug name | OMEPRAZOLE, ESOMEPRAZOLE, LANSOPRAZOLE, DEXLANSOPRAZOLE, PANTOPRAZOLE, RABEPRAZOLE |
| SSRI/SNRI | Generic drug name | CITALOPRAM, ESCITALOPRAM, FLUOXETINE, FLUVOXAMINE, PAROXETINE, SERTRALINE, VILAZODONE, DULOXETINE, VENLAFAXINE, LEVOMILNACIPRAN  DESVENLAFAXINE |

**Table S2.** Characteristics of patients with new onset AFib and history of cancer in SEER-Medicare registry from 2012 to 2018 (Stroke)

|  | **Overall (N=18388)** | **Stroke (N=523)** | **Non-stroke (N=17865)** |
| --- | --- | --- | --- |
| **Demographics** |  |  |  |
| Index age (Mean, SD) | 76.59 (7.13) | 78.55 (7.72) | 76.53 (7.11) |
| Female | 8483 (46.13) | 270 (51.63) | 8213 (45.97) |
| Race/ethnicity |  |  |  |
| Non-Hispanic White | 15650 (85.11) | 432 (82.60) | 15218 (85.18) |
| Non-Hispanic Black | 1134 (6.17) | 38 (7.27) | 1096 (6.13) |
| Others | 1604 (8.72) | 53 (10.13) | 1551 (8.68) |
| Region |  |  |  |
| Midwest | 1604 (8.72) | 34 (6.50) | 1570 (8.79) |
| Northeast | 7195 (39.13) | 216 (41.30) | 6979 (39.07) |
| South | 3263 (17.75) | 93 (17.78) | 3170 (17.74) |
| West | 6326 (34.40) | 180 (34.42) | 6146 (34.40) |
| Medicaid eligible | 2007 (10.91) | 63 (12.05) | 1944 (10.88) |
| Urbanicity |  |  |  |
| Metropolitan | 15895 (86.44) | 459 (91.98) | 15436 (91.48) |
| Micropolitan | 1478 (8.04) | 40 (8.02) | 1438 (8.52) |
| Unknown | 1015 (5.52) |  |  |
| **Socioeconomic status (Census Tract)** |  |  |  |
| Household median income (Median, IQR) (N=18086) | 62122 (45499-84935) | 63092 (47253-85846) | 62096 (45482-84914) |
| Percentage of non-high school graduates (Median, IQR) (N=18090) | 9.53 (5.20-16.56) | 9.28 (5.07-15.45) | 9.54 (5.20-16.58) |
| **Cancer characteristics** |  |  |  |
| Time from cancer diagnosis to the onset of AFib (month, Median, IQR) | 17 (2-40) | 28 (9-51) | 16 (2-40) |
| Cancer type |  |  |  |
| Breast | 5643 (30.69) | 227 (43.40) | 5416 (30.32) |
| Lung | 6165 (33.53) | 106 (20.27) | 6059 (33.92) |
| Prostate | 6580 (35.78) | 190 (36.33) | 6390 (35.77) |
| Active cancer | 4629 (25.17) | 128 (24.47) | 4501 (25.19) |
| Cancer grade |  |  |  |
| I | 2583 (14.05) | 83 (15.87) | 2500 (13.99) |
| II | 6583 (35.80) | 198 (37.86) | 6385 (35.74) |
| III | 5955 (32.39) | 160 (30.59) | 5795 (32.44) |
| Others | 3267 (17.77) | 82 (15.68) | 3185 (17.83) |
| Cancer stage |  |  |  |
| In situ | 883 (4.80) | 839 (4.97) | 44 (8.87) |
| Local | 10782 (58.64) | 311 (62.70) | 10471 (61.99) |
| Regional | 3941 (21.43) | 100 (20.16) | 3841 (22.74) |
| Distant | 1781 (9.69) | 41 (8.27) | 1740 (10.30) |
| Unknown/missing | 1001 (5.44) |  |  |
| **Cancer treatment** |  |  |  |
| Potential interacting antineoplastic agents | 3964 (21.56) | 139 (26.58) | 3825 (21.41) |
| Radiation | 2419 (13.16) | 51 (9.75) | 2368 (13.25) |
| Surgery | 1692 (9.20) | 54 (10.33) | 1638 (9.17) |
| **Individual comorbidities** |  |  |  |
| HTN | 13259 (72.11) | 384 (73.42) | 12875 (72.07) |
| CHF | 2343 (12.74) | 54 (10.33) | 2289 (12.81) |
| Diabetes | 5621 (30.57) | 188 (35.95) | 5433 (30.41) |
| Prior stroke | 1542 (8.39) | 96 (18.36) | 1446 (8.09) |
| Prior vascular diseases | 4385 (23.85) | 136 (26.00) | 4249 (23.78) |
| Prior major bleeding | 3843 (20.90) | 86 (16.44) | 3757 (21.03) |
| Renal diseases | 3631 (19.75) | 104 (19.89) | 3527 (19.74) |
| Liver diseases | 1635 (8.89) | 38 (7.27) | 1597 (8.94) |
| Alcohol use disorders | 526 (2.86) | 13 (2.49) | 513 (2.87_ |
| Asthma/COPD | 6323 (34.39) | 119 (22.75) | 6204 (34.73) |
| Hematological disorders | 5664 (30.80) | 139 (26.58) | 5525 (30.93) |
| Dementia | 1044 (5.68) | 33 (6.31) | 1011 (5.66) |
| Depression | 2650 (14.41) | 76 (14.53) | 2574 (14.41) |
| Acute kidney diseases | 1183 (6.43) | 31 (5.93) | 1152 (6.45) |
| Thrombocytopenia | 1120 (6.09) | 22 (4.21) | 1098 (6.15) |
| Peptic ulcer diseases | 279 (1.52) | -- | -- |
| **Medications** |  |  |  |
| Antiplatelet/NSAIDs | 2957 (16.08) | 93 (17.78) | 2864 (16.03) |
| ACE inhibitors/ARBs | 4691 (25.51) | 172 (32.89) | 4519 (25.30) |
| CCB | 2952 (16.05) | 115 (21.99) | 2837 (15.88) |
| Beta blockers | 4135 (22.49) | 155 (29.64) | 3980 (22.28) |
| Antiarrhythmic medications | 915 (4.98) | 15 (2.87) | 900 (5.04) |
| Diuretics | 3235 (17.59) | 104 (19.89) | 3131 (17.53) |
| Statin | 4856 (26.41) | 152 (29.06) | 4704 (26.33) |
| PPIs | 2844 ((15.47) | 80 (15.30) | 2764 (15.47) |
| SSRIs/SNRIs | 1979 (10.76) | 63 (12.05) | 1916 (10.72) |
| CHA_2_DS_2_-VASc score  1  2  3  4  5  6  7  8  9 | 1674 (9.10)  3185 (17.32)  4437 (24.13)  4440 (24.15)  2638 (14.35)  1319 (7.17)  506 (2.75)  153 (0.83)  36 (0.20) | 32 (6.12)  73 (13.96)  104 (19.89)  123 (23.52)  98 (18.74)  54 (10.33)  28 (5.35)  9 (1.72)  2 (0.38) | 1642 (9.19)  3112 (17.42)  4333 (24.25)  4317 (24.16)  2540 (14.22)  1265 (7.08)  478 (2.68)  144 (0.81)  34 (0.19) |
| **Outcome** |  |  |  |
| Ischemic stroke | 523 (2.84) |  |  |
| Major bleeding | 221 (1.20) |  |  |

--: suppressed due to cell size <11 or because cell size <11 can be derived from other cells
AFib Atrial Fibrillation. SD Standard Deviation. IQR Interquartile Range. HTN hypertension. CHF congestive heart failure. COPD Chronic obstructive pulmonary disease. ACE Angiotensin-converting enzyme. ARB Angiotensin receptor blockers. CCB Calcium Channel Blockers. PPI Pump Proton Inhibitors. SSRI Selective serotonin reuptake inhibitors. SNRI Serotonin and norepinephrine reuptake inhibitors. NSAIDs non-steroidal anti-inflammatory drugs.

**Table S3.** Characteristics of patients with new onset AFib and history of cancer in SEER-Medicare registry from 2012 to 2018 (Bleeding)

|  | **Overall (N=18838)** | **Bleeding (N=221)** | **Non-bleeding (N=17865)** |
| --- | --- | --- | --- |
| **Demographics** |  |  |  |
| Index age (Mean, SD) | 76.59 (7.13) | 78.98 (7.15) | 76.56 (7.13) |
| Female | 8483 (46.13) | 101 (45.70) | 8382 (46.14) |
| Race/ethnicity |  |  |  |
| Non-Hispanic White | 15650 (85.11) | 182 (82.35) | 15468 (85.14) |
| Non-Hispanic Black | 1134 (6.17) | 13 (5.88) | 1121 (6.17) |
| Others | 1604 (8.72) | 26 (11.76) | 1578 (8.69) |
| Region |  |  |  |
| Midwest | 1604 (8.72) | -- | -- |
| Northeast | 7195 (39.13) | 94 (42.53) | 7101 (39.09) |
| South | 3263 (17.75) | 42 (19.00) | 3221 (17.73) |
| West | 6326 (34.40) | 75 (33.94) | 6251 (34.41) |
| Medicaid eligible | 2007 (10.91) | 25 (11.31) | 1982 (10.91) |
| Urbanicity |  |  |  |
| Metropolitan | 15895 (86.44) | 204 (94.88) | 15691 (91.45) |
| Micropolitan | 1478 (8.04) | 11 (5.12) | 1467 (8.55) |
| Unknown | 1015 (5.52) |  |  |
| **Socioeconomic status (Census Tract)** |  |  |  |
| Household median income (Median, IQR) | 62122 (45499-84935) | 72723 (52492-93610) | 62051 (45435-84839) |
| Percentage of non-high school graduates (Median, IQR) (N=18086) | 9.53 (5.20-16.56) | 7.65 (4.33-14.73) | 9.55 (5.21-16.60) |
| **Cancer characteristics** |  |  |  |
| Time from cancer diagnosis to the onset of AFib (month, Median, IQR) (N=18090) | 17 (2-40) | 25 (6-46) | 16 (2-40) |
| Cancer type |  |  |  |
| Breast | 5643 (30.69) | 84 (38.01) | 5559 (30.60) |
| Lung | 6165 (33.53) | 57 (25.79) | 6108 (33.62) |
| Prostate | 6580 (35.78) | 80 (36.20) | 6500 (35.78) |
| Active cancer | 4629 (25.17) | 55 (24.89) | 4574 (25.18) |
| Cancer grade |  |  |  |
| I | 2583 (14.05) | 37 (16.74) | 2546 (14.01) |
| II | 6583 (35.80) | 67 (30.32) | 6516 (35.87) |
| III | 5955 (32.39) | 74 (33.48) | 5881 (32.37) |
| Others | 3267 (17.77) | 43 (19.46) | 3224 (17.75) |
| Cancer stage |  |  |  |
| In situ | 883 (4.80) | 13 (6.22) | 870 (5.06) |
| Local | 10782 (58.64) | 137 (65.55) | 10645 (61.97) |
| Regional | 3941 (21.43) | 33 (15.79) | 3908 (22.75) |
| Distant | 1781 (9.69) | 26 (12.44) | 1755 (10.22) |
| Unknown/missing | 1001 (5.44) |  |  |
| **Cancer treatment** |  |  |  |
| Potential interacting antineoplastic agents | 3964 (21.56) | 49 (22.17) | 3915 (21.55) |
| Radiation | 2419 (13.16) | 30 (13.57) | 2389 (13.15) |
| Surgery | 1692 (9.20) | 20 (9.05) | 1672 (9.20) |
| **Individual comorbidities** |  |  |  |
| HTN | 13259 (72.11) | 167 (75.57) | 13092 (72.06) |
| CHF | 2343 (12.74) | 32 (14.48) | 2311 (12.72) |
| Diabetes | 5621 (30.57) | 66 (29.86) | 5555 (30.58) |
| Prior stroke | 1542 (8.39) | 53 (23.98) | 1489 (8.20) |
| Prior vascular diseases | 4385 (23.85) | 63 (28.51) | 4322 (23.79) |
| Prior major bleeding | 3843 (20.90) | 72 (32.58) | 3771 (20.76) |
| Renal diseases | 3631 (19.75) | 34 (15.38) | 3597 (19.80) |
| Liver diseases | 1635 (8.89) | -- | -- |
| Alcohol use disorders | 526 (2.86) | -- | -- |
| Asthma/COPD | 6323 (34.39) | 69 (31.22) | 6254 (34.43) |
| Hematological disorders | 5664 (30.80) | 82 (37.10) | 5582 (30.73) |
| Dementia | 1044 (5.68) | 24 (10.86) | 1020 (5.61) |
| Depression | 2650 (14.41) | 43 (19.46) | 2607 (14.35) |
| Acute kidney diseases | 1183 (6.43) | 12 (5.43) | 1171 (6.45) |
| Thrombocytopenia | 1120 (6.09) | 12 (5.43) | 1108 (6.10) |
| Peptic ulcer diseases | 279 (1.52) | -- | -- |
| **Medications** |  |  |  |
| Antiplatelet/NSAIDs | 2957 (16.08) | 34 (15.38) | 2923 (16.09) |
| ACE inhibitors/ARBs | 4691 (25.51) | 53 (23.98) | 4638 (25.53) |
| CCB | 2952 (16.05) | 34 (15.38) | 2918 (16.06) |
| Beta blockers | 4135 (22.49) | 46 (20.81) | 4089 (22.51) |
| Antiarrhythmic medications | 915 (4.98) | -- | -- |
| Diuretics | 3235 (17.59) | 35 (15.84) | 3200 (17.61) |
| Statin | 4856 (26.41) | 57 (25.79) | 4799 (26.42) |
| PPIs | 2844 ((15.47) | 32 (14.48) | 2812 (15.48) |
| SSRIs/SNRIs | 1979 (10.76) | 35 (15.84) | 1944 (10.70) |
| HAS-BLED score  1  2  3  4  5  6  7  8 | 3824 (20.80)  6213 (33.79)  5046 (27.44)  2354 (12.80)  749 (4.07)  183 (1.00)  18 (0.10)  1 (0.01) | 40 (18.10)  64 (28.96)  62 (28.05)  35 (15.84)  19 (8.60)  1 (0.45)  0 (0.00)  0 (0.00) | 3784 (20.83)  6149 (33.85)  4984 (27.43)  2319 (12.76)  730 (4.02)  182 (1.00)  18 (0.10)  1 (0.01) |
| **Outcome** |  |  |  |
| Ischemic stroke | 523 (2.84) |  |  |
| Major bleeding | 221 (1.20) |  |  |

--: suppressed due to cell size <11 or because cell size <11 can be derived from other cells
AFib Atrial Fibrillation. SD Standard Deviation. IQR Interquartile Range. HTN hypertension. CHF congestive heart failure. COPD Chronic obstructive pulmonary disease. ACE Angiotensin-converting enzyme. ARB Angiotensin receptor blockers. CCB Calcium Channel Blockers. PPI Pump Proton Inhibitors. SSRI Selective serotonin reuptake inhibitors. SNRI Serotonin and norepinephrine reuptake inhibitors. NSAIDs non-steroidal anti-inflammatory drugs.

**Overview of machine learning models used in our study**

**Elastic net logistic regression** is the ML algorithm that combines multivariable logistic regression with LASSO and ridge regression penalty terms. For example, consider a ridge regression model

$$\sum_{i=1}^{n} ({y_{i}- \beta_{0}- \sum_{j=1}^{p} \beta_{i}x_{ij})}^{2}+ A=RSS+ A$$

where A= $\lambda\sum_{j=1}^{p} \beta_{j}^{2}$ is a shrinkage penalty for ridge regression and A= $\lambda\sum_{j=1}^{p} \left| \beta_{j} \right|$ for LASSO. These penalty terms shrink the model coefficients to towards zero to reduce overfitting.^1^ Thus, the models have variable selection property and achieve a higher performance.

**Random forests and XGBoost** are aggregated decision tree models with substantially improved predictive performance.^1^ XGBoost improves the accuracy of individual decision trees by building many trees sequentially and weighs the difficult-to-predict cases in a tree to a greater degree. XGBoost also increases the speed of the algorithm and includes penalty terms to avoid model overfitting. On the other hand, random forests builds each tree separately based on a random sample of the training data. For each split, a random number of predictor variables are available, which results in trees that are different from each other. Therefore, the final model contains thousands of individual decision trees, with each of them combining to make predictions on new patients. When fitting the XGBoost and random forest models, we will specify the number of trees, depth of trees, learning rate, and the number of predictor variables available at each split using 10-fold cross-validation in the training data.

**Support vector machines** **(SVMs)**: SVMs puts the data into hyperplane and creates linear or non-linear decision boundary that maximize the margin between the classes of the outcomes using kernels.^1^ The optimal value of the cost penalty (misclassification) will be determined using 10-fold cross-validation in the training data.

**Neural networks (NNs)**: NNs mimic how neurons works by combining of individual neuron-like units that take the predictor variables as inputs (input layer), combine them in hidden layers, process them through activation functions, and then output predictions (output layers).^1^ In this study, we will use a feed-forward multi-layer perceptron NN. We will specify the number of hidden layers, size of each hidden layer, learning rate, and decay using ten-fold cross-validation in the training data.

**References**

1. James G, Witten D, Hastie T, Tibshirani R. *An Introduction to Statistical Learning with Applications in R.* Springer; 2013.
